# Effectiveness of functional ingredients to enhance gill disease in Atlantic salmon (Salmo salar, L.)

**DOI:** 10.1371/journal.pone.0304112

**Published:** 2024-06-20

**Authors:** Matteo Vitale, Eirik Hoel, Muhammad Naveed Yousaf, Martha Amalie Kambestad, Julia Mullins, Leidy Lagos, Kjetil Berge, Charles McGurk, Daniela Maria Pampanin

**Affiliations:** 1 Department of Chemistry, Bioscience and Environmental Engineering, University of Stavanger, Stavanger, Norway; 2 Skretting Aquaculture Innovation, Stavanger, Norway; 3 Skretting AS, Stavanger, Norway; 4 Bremnes Seashore, Bremnes, Norway; Benha University, EGYPT

## Abstract

The development and application of functional feed ingredients represents a great opportunity to advance fish growth and health, boost the immune system, and induce physiological benefits beyond those provided by traditional feeds. In the present study, we looked at the feasibility of *in vitro* methods for screening the qualities of functional feed ingredients using the fish cell line RTgill-W1, which has never been used in fish nutrition, and the culture of *Paramoeba perurans*. Five functional feed ingredients (arginine, β-glucan, vitamin C, and two phytogenic feed additives) were selected to investigate their effects on cell viability and reactive oxygen species production. Three of the selected ingredients (arginine and two phytogenic feed additives) were additionally tested to assess their potential amoebicidal activity. As these functional ingredients are the core of a commercially available feed (Protec Gill, Skretting AS), their beneficial effects were further assessed in a field trial in fish affected by complex gill disease. Here, the analyzed parameters included the evaluation of macroscopic and histopathological gill conditions, pathogen detections, and analyses of plasma parameters. RTgill-W1 cell line assays were a good tool for screening functional ingredients and provided information about the optimal ingredient concentration ranges, which can be helpful for adjusting the concentrations in future feed diets. Through the culture of *P*. *perurans*, the tested ingredients showed a clear amoebicidal activity, suggesting that their inclusions in dietary supplements could be a viable way to prevent microbial infections. A three-week period of feeding Protec Gill slowed the disease progression, by reducing the pathogen load and significantly improving gill tissue conditions, as revealed by histological evaluation. The use of diets containing selected functional ingredients may be a feasible strategy for preventing or mitigating the increasingly common gill diseases, particularly in cases of complex gill disease, as documented in this study.

## Introduction

In the last decades the expansion of the aquaculture industry has been accompanied by a significant rise in fish feed production, which has led increased pressure on marine supplies such as fish meal and fish oil, resulted in low availability and high prices [1]. Reducing reliance on marine commodities by extending the use of alternative ingredients will improve the future sustainability of the aquaculture practice. The application of functional feed ingredients represents a great opportunity to advance fish growth and health, boost the immune system, and induce physiological benefits beyond those provided by traditional feed [2]. Numerous dietary supplements with immunomodulatory properties can be used to stimulate the immune system of fish [3, 4], such as single amino acids like arginine [5, 6], vitamins [7, 8], β-glucans [9–11], and phytogenic feed additives (PFAs) [12–15], which use has been recently introduced in aquaculture. Functional feed ingredients may also play an important role in lowering disease susceptibility [16, 17].

In European salmon-producing countries like Norway, Scotland and Ireland, gill diseases have become one of the most significant health challenges for the aquaculture industry [18–20]. They can be classified as either simple or complex/multifactorial gill disease, based on a presumption of single or multiple causes and infectious agents involved in the pathogenesis [21, 22]. Typically, the term multifactorial gill disease or complex gill disease (CGD) is used to describe the type of gill illness in which multiple causes can be observed simultaneously and there are no obvious primary causal agents [21, 23–26]. CGD encompasses syndromes referred to as proliferative gill inflammation (PGI) and proliferative gill disease (PGD) [26]. PGI is a pathology-based diagnosis first described in Norway, in which gills mainly present a combination of lamellar vascular changes, inflammation, cell death, epithelial and mucous hyperplasia [27]. PGD has been used as a non-specific term for the examination of gross lesions in the salmon gills [26], and as a general descriptive term for proliferative changes in the gill epithelium [28].

The aim of the present study was to evaluate the feasibility of *in vitro* methods for screening qualities of functional feed ingredients, using the fish cell line RTgill-W1, which has never been used in fish nutrition, and the culture of *Paramoeba perurans*. As the selected functional ingredients are the core of a commercially available feed (Protec Gill, Skretting AS), their beneficial effects were further assessed in a field trial in fish affected by CGD.

## Materials and methods

### Functional feed ingredients

Five functional feed ingredients were used in this study: arginine, β-glucan, vitamin C, and two PFAs. β-glucan and PFAs are proprietary composition of Skretting ARC and encompassing confidential information. All stock solutions were freshly prepared and used immediately for the assays (see Table 1 in S1 File).

### RTgill-W1 cell culture

RTgill-W1 cells (American Type Culture Collection No. CRL-2523, commercially available), obtained from gill explants of adult rainbow trout (*Oncorhynchus mykiss*), were cultured using 75 cm^2^ tissue culture flasks (19°C, w/o CO_2_, in the dark), in Leibovitz’s (L-15) media, containing 10% (v/v) of fetal bovine serum (Biowest, Nuaillé, France), 100 U/mL penicillin and 100 μg/mL streptomycin (Gibco, NY, USA) [29]. Confluent cells were exposed in 96-well plates (3 x 10^4^ cells per well) to a range of concentrations of the selected functional feed ingredients (S1A Table in S1 File). For each concentration seven replicates were tested (n = 7).

#### Cell viability assay

Cell viability was assessed using a fluorometric assay as described by [30], with small adjustments. Confluent cells were exposed for 24 h to each ingredient. Control (cells with only L-15), blank (only L-15) and positive control (hydrogen peroxide, H_2_O_2_, 100 μM, diluted in L-15) were also tested. After incubation, the exposure solution was removed and the resazurin dye (484 μM, AlamarBlueTM, ThermoFisher Scientific) was added (1:10 ratio with L-15). The ratio of viable cells was quantified using a microplate reader (SpectraMax Paradigm Multi-Mode), which measured the fluorescence of resorufin at excitation and emission wavelengths of 530 and 590 nm, respectively. Relative fluorescence units (RFUs) were normalized to the control.

#### Reactive oxygen species production assay

Reactive oxygen species (ROS) production was measured by applying a modified version of the protocol described by [31]. Once the cell monolayer was formed, the media was carefully removed, followed by a wash with phosphate buffer saline (PBS). The 2’,7’- dichorodihydrofluorescein diacetate (H2DCF-DA) probe was used as ROS production indicator. It was freshly prepared and 100 μL of 10 mM solution were added in each well (except for the blank), following an incubation time of 30 min. Afterwards, the probe was removed and two additional washes with PBS were done. Then, RTgill-W1 cells were exposed for 60 min to each ingredient. Control (cells with only PBS), blank (only PBS) and positive control (H_2_O_2_, 100 μM, diluted in PBS) were also tested. The fluorescence emitted due to the oxidation of H2DCF-DA was read using the microplate reader at excitation and emission wavelengths of 485 and 528 nm, respectively. RFUs were normalized to the control.

### Amoebae survival *in vitro* testing

The culture of *Paramoeba perurans* was obtained from ILAB in cell culture flasks containing malt yeast broth (MYB; malt and yeast extract in 75% seawater). The amoebae were kept in an incubator at 15°C and sub-cultured in MYB weekly or biweekly, depending on the density of the amoebae and the density of co-occurring bacteria in the cultures, which the amoebae depend upon [32]. Prior to the test, amoebae attached to the flask or floating were concentrated by a centrifugation step at 1000g for 15 min before being resuspended in 5 mL aseptic seawater (ASW). Pelleted amoebae were then mixed with an ingredient (S1B Table in S1 File) in a 24 cell-well plate. Amoebae culture (1 x 10^5^ per well) were exposed for 24 h to each ingredient in seawater, in addition to the control of seawater only. Then amoebae were pelleted at 1 x 10^4^ cells and resuspended in 200 μL of seawater containing 10 μg/mL fluorescein diacetate (ThermoFisher Scientific, Prague, Czech Republic; life stain) and with 1 μg/mL propidium iodine (ThermoFisher Scientific, Prague, Czech Republic; dead cells) in the dark, at room temperature, for 10 min. Live and dead cells were quantified using a flow cytometer (BD FACSCanto II, BD Biosciences).

### Field trial

The field feeding trial was conducted in a commercial farming site on the west coast of Norway (Bremnes, Norway), where the functional diet Protec Gill (Skretting AS) was tested in comparison with a standard high performance commercial feed (Express 2500, Skretting AS). The trial started at the end of September 2020 and was conducted over a period of three weeks. Fish (Atlantic salmon, *Salmo salar*) were initially divided into four cages (see Table 2 in S1 File). For the whole experimental period (from T_0_ to T_3_), two cages (1 and 2) were fed Protec Gill, whereas the two other cages (3 and 4) received Express 2500. An air-pressure driven central feeding system was used for delivering feed to each cage through polyethylene-hoses. All feed used (9 mm diameter) were formulated and produced by extruding technology according to standard procedures in a commercial fish factory (Skretting, Stavanger, Norway) (see Table 3 in S1 File). One month prior to the trial, gills from ten fish were subjected to histopathological analysis by Fish Vet Group AS (Oslo, Norway), providing findings compatible with CGD, through evidence of epithelial and mucous hyperplasia, hyperplastic multifocal gill inflammation, necrotic/apoptotic epithelial cells, presence of epitheliocystis and amoebae. In addition, the presence of pathogens such as *Paramoeba perurans*, *Ca*. *Branchiomonas cysticola*, *Paranucleospora theridion* (syn. *Desmozoon lepeophtherii*), and salmon gill poxvirus (SGPV) in the gill tissue were confirmed by qPCR analysis.

#### Field trial design and husbandry

Prior to the trial, all cages were deloused 4–6 times using tempered seawater with Thermolicer or Optilice technology (see Table 4 in S1 File). Only cage 2 and 3 were treated using Thermolicer during the trial period (at T_2_). Four-five days before delousing, fish did not receive feed. All pens were cleaned using high pressure seawater according to standard procedures, approximately once a week, before and during the trial period.

Feeding and mortality were registered in a production management system (Mercatus Farmer, Scale Aquaculture AS, Norway). Dead fish were registered and removed from each cage daily.

The weight of the fish was estimated using a production management system based on initial number of fish and weight, feeding and mortality. Specific growth rate (SGR) was calculated based on data from AKVA Fishtalk production management using the following equation:

$$\text{Specific}\;\text{growth}\;\text{rate}(\text{SGR},\;\%\;\text{growth}\;\text{per}\;\text{day})=\left({\left(\frac{\text{FBW}}{\text{IBW}}\right)}^{1/\text{Days}}-1\right)\times 100$$

This research was carried out in strict accordance with Norwegian aquaculture production and animal welfare regulations (Forskrift om drift av akvakulturanlegg 34. Avlivning av fisk). No approval was necessary as commercial farm samplings in the field do not need to be approved by the Norwegian Food Safety Authority (FOTS), and no live animals were harmed since all testing were done post-mortem. Fish were randomly caught using a large seine and euthanized with an overdose of benzocaine (Benzoak vet., ACD Pharmaceuticals AS, Leknes, Norway), with every effort made to reduce the pain.

#### Macroscopic score of gills

Amoebic gill disease (AGD) scoring was calculated according to [33]: score 0 (clear), no sign of infection and healthy red colour; score 1 (very light), one white spot, light scarring or undefined necrotic; score 2 (light), 2–3 spots and small mucous patch; score 3 (moderate), established thickened mucous patch or spot grouping up to 20% of gill area; score 4 (advanced), established lesions covering up to 50% of gill area; score 5 (heavy), extensive lesions covering most of the gill surface.

The PGD was scored according to a system developed by Mowi Scotland. Score 0: normal gills and no pathological changes; score 1: very slight thickening or very few lamellae affected; score 2: frequent thickening, but tips only; score 3: almost all lamellae have thickened tips, and some have thickenings progressing to 50% of the length of the lamellae; score 4: most lamellae have thickenings progressing to more than 50% of the length of the lamellae; score 5: almost all lamellae are thickened along entire length.

Fish were analysed per diet group for each sampling point (T_0_, n = 20; T_3_, n = 40).

#### Pathogen detection

Gill tissue (2 x 2 x 5 mm) from the middle of the second arch was placed in RNA-later and shipped on ice to Patogen AS (Ålesund, Norway) to perform qPCR analysis of targeting *P*. *perurans*, *Ca*. *B*. *cysticola*, *P*. *theridion* and SGPV. The qPCR analysis was validated to ISO17025 standards by Patogen AS. Samples were defined as positive when having cycles to threshold (Ct) value lower than 37.0. Elongation factor 1α (EF1α) served as an internal reference gene for all qPCR analysis performed [34]. Results were expressed in Ct of PCR, a relative value that represents the cycle number at which the amount of amplified DNA reaches the threshold level. Low pathogen load was represented by high Ct value.

Fish were analysed per diet group for each sampling point (T_0_, n = 20; T_3_, n = 30).

#### Gill histology

**Fig 1:**
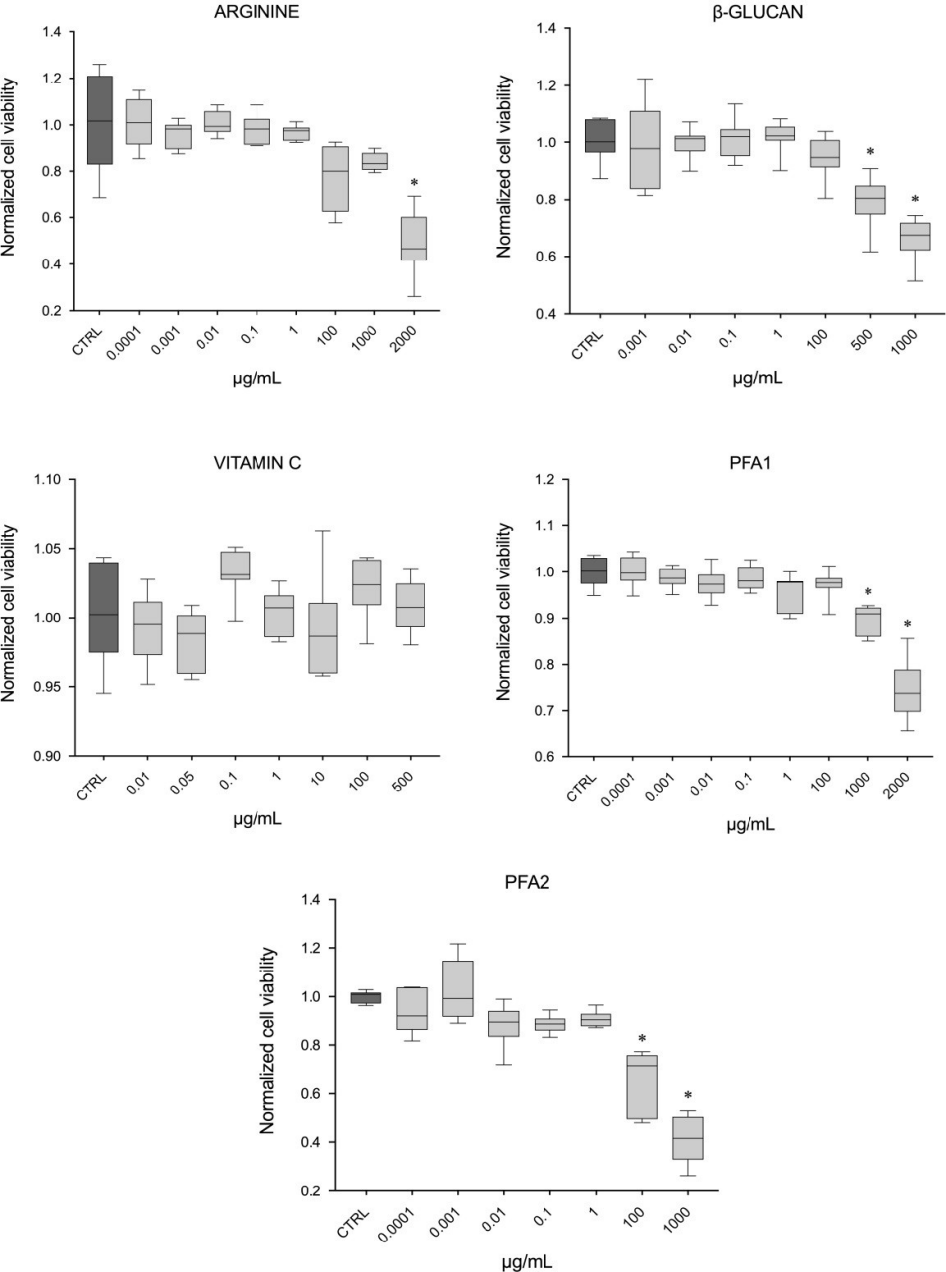
Cell viability. *In vitro* cell viability assay, results reported per ingredient: (A) arginine, (B) β-glucan, (C) vitamin C, (D) phytogenic feed additive 1, (E) phytogenic feed additive 2. Values are expressed as μg/mL and reported as median ± standard deviation, normalized to the control (CTRL); (n = 7), * = p < 0.05 (compared to the control).

**Fig 2:**
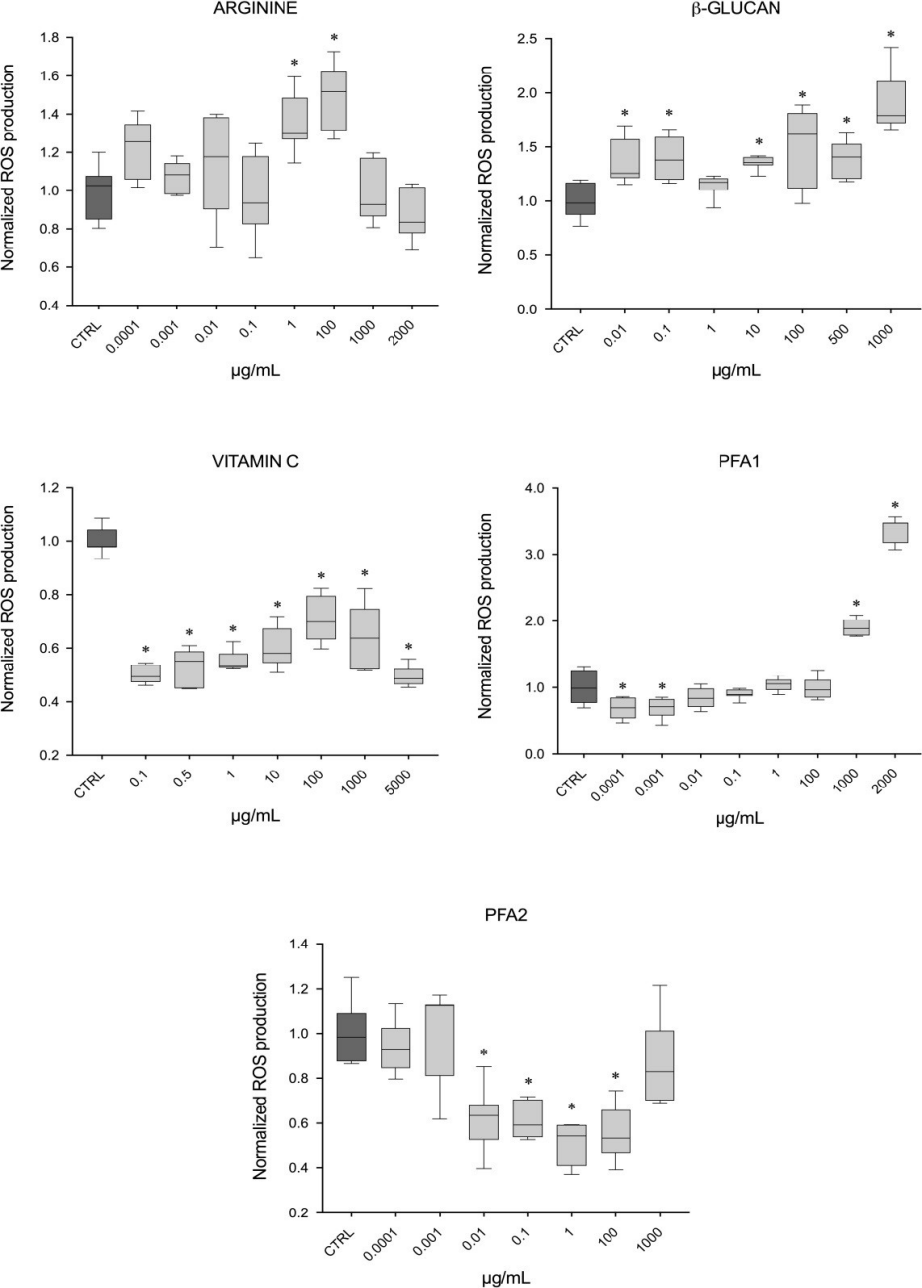
Reactive oxygen species production. ROS production assay, results reported per ingredient: (A) arginine, (B) β-glucan, (C) vitamin C, (D) phytogenic feed additive 1, (E) phytogenic feed additive 2. Values are expressed as μg/mL and reported as median ± standard deviation, normalized to the control (CTRL); (n = 7), * p < 0.05 (compared to the control).

Gill tissue from the second gill arch was fixed in 10% neutral phosphate buffered formalin (VWR, International AS, Oslo, Norway) for histopathological evaluation. Samples were processed, sectioned at 2 μm and stained with hematoxylin and eosin [35]. Sections were evaluated for tissue changes using a light microscope according to a scoring system adapted from [36] (see Table 5 in S1 File). Representative images of the scoring system are reported in Fig 1 in S1 File. Pathogen such as amoeba and cysts such as epitheliocystis, epithelial hyperplasia and lamellar fusion, epithelial and mucous hyperplasia, and necrosis were identified (see Fig 2 in S1 File). The slides were digitized using a slide scanner (Panoramic SCAN II, 3 Dhishtech) for images.

Fish were analysed per diet group for each sampling point (T_0_, n = 20; T_3_, n = 30).

#### Plasma parameters

Blood samples were taken from tail vessels (vena/arteria caudalis) by Vacuette containers containing lithium-heparin. Samples were centrifuges immediately after collection at 4000 g for 6 min. Then the plasma was frozen and stored at -80°C until further analysis. Lysozyme activity was analysed according to [37] and expressed as U/mol, whereas C-reactive protein (CRP) analysis was carried out using a Konelab 30i (ThermoFisher Scientific) and expressed as mg/L.

Fish were analysed per diet group for each sampling point (T_0_, n = 20; T_3_, n = 30).

### Statistical analysis

GraphPad prism 9 (GraphPad software LLC, San Diego, USA) was used for the statistical analysis. Normality distribution of the data was assessed with Shapiro-Wilks test. Normally distributed data were analysed using one-way ANOVA. When the data were not normally distributed, the non-parametric Kruskal-Wallis test was used. A post-hoc (Tukey’s multiple comparisons test) was performed to identify which groups differ from each other. Asterisks denote the level of a statistical significance (* = p < 0.05).

## Results

### *In vitro* study

#### Cell viability assay

A significant decrease in cell viability was observed only at high tested concentrations (Fig 1): at 2000 μg/mL for arginine; at 500 and 1000 μg/mL for β-glucan; at 1000 and 2000 μg/mL for PFA 1, and at 100 and 1000 μg/mL for PFA 2. Only vitamin C had values similar to the control at all tested concentrations.

#### Reactive oxygen species production assay

A significant decrease in ROS production was recorded in cells exposed to vitamin C at all tested concentrations (from 0.1 to 5000 μg/mL), to PFA 1 at the lowest concentrations (0.0001 and 0.001 μg/mL) and PFA 2 in a range of concentrations from 0.01 to 100 μg/mL (Fig 2). A significant increase in ROS production was observed after exposure to arginine (1 and 100 μg/mL), PFA 1 at the highest concentrations (1000 and 2000 μg/mL), and β-glucan at all tested concentrations (from 0.01 to 1000 μg/mL).

#### Amoebae survival *in vitro* testing

**Fig 3:**
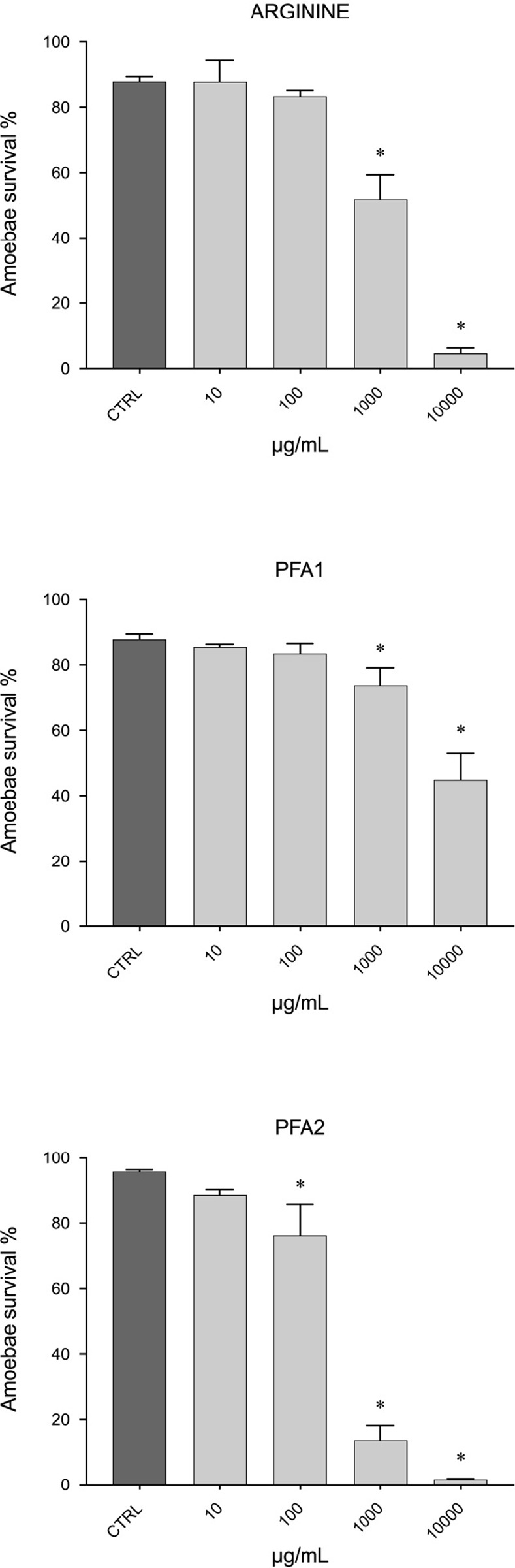
Amoebae survival. Amoebae (*Paramoeba perurans*) survival testing, results reported per ingredient: (A) arginine, (B) phytogenic feed additive 1, (C) phytogenic feed additive 2. Values are expressed as amoebae survival percentage (%) and reported as mean ± standard deviation, compared to the control (CTRL); (n = 3), * p < 0.05 (compared to the control).

A significant decrease in the survival of *P*. *perurans* was observed after exposure to arginine (10000 μg/mL and 1000 μg/mL), PFA 1 (10000 μg/mL, 1000 μg/mL, and 100 μg/mL) and PFA 2 (10000 μg/mL and 1000 μg/mL) (Fig 3).

### Field trial

#### Fish growth and survival

There were no statistical differences in the feeding rate and the calculated SGR between groups during the study. The mean SGR percentage was higher in the Protec Gill group (0.73%) compared to the Express group (0,61%), although without significant differences.

Mortality was recorded for a broad period of time (from 3 weeks before the trial (T_-3_) until 3 weeks after the trial (T_6_)) and the calculated average mortality ranged from 0.1% to 0.36% during the whole period (see Fig 3 in S1 File).

#### Macroscopic gill score

**Fig 4:**
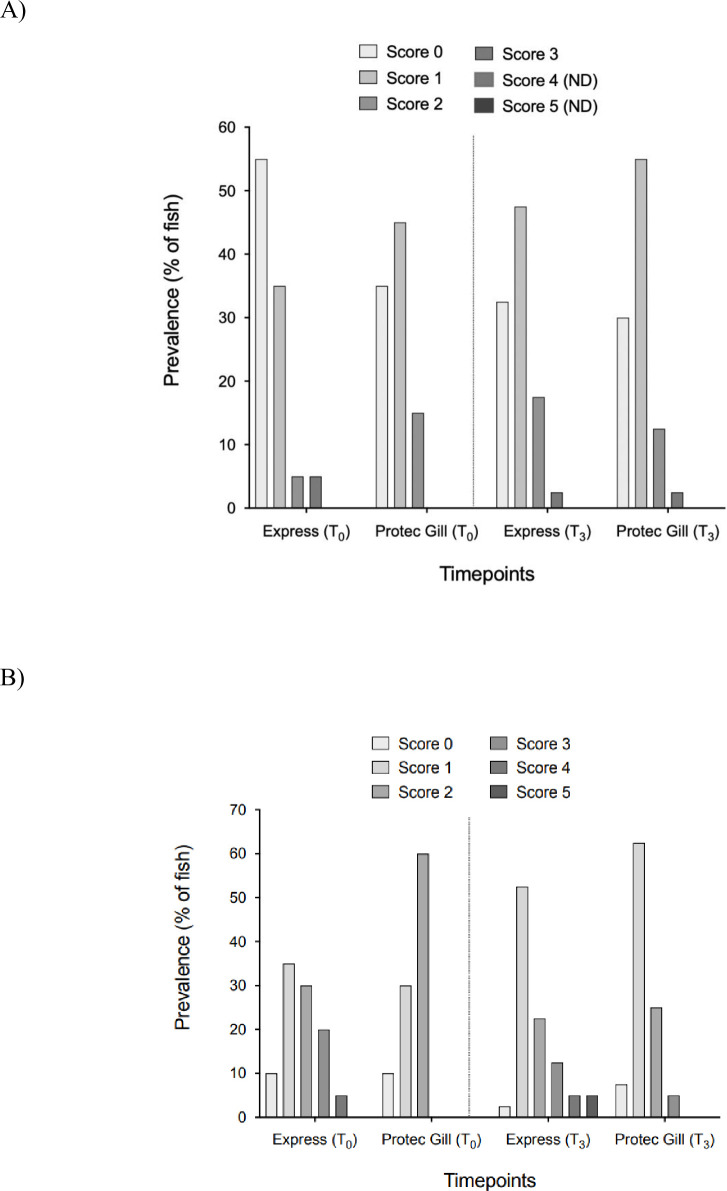
Macroscopic gill score. Amoebic gill disease score (A) and proliferative gill disease score (B) at the beginning of the study (T_0_) and at the end (T_3_) in Atlantic salmon gill tissue. Results are reported as prevalence percentage of fish with a specific score; n = 20 (T_0_), n = 40 (T_3_).

No statistical differences between diet groups were found either in AGD-score or PGD-score after three weeks of administration (Fig 4). However, a progression for both scores was observed at T_3_ in both groups. For AGD-score, Protec Gill group showed scores from 0 to 2 at T_0_, and from 0 to 3 at T_3_ (Fig 4A). Whereas for PGD score, Express group showed all the five scores at T_3_, and Protec Gill group had score from 0 to 4 at T_3_.

#### Pathogen detection

**Fig 5:**
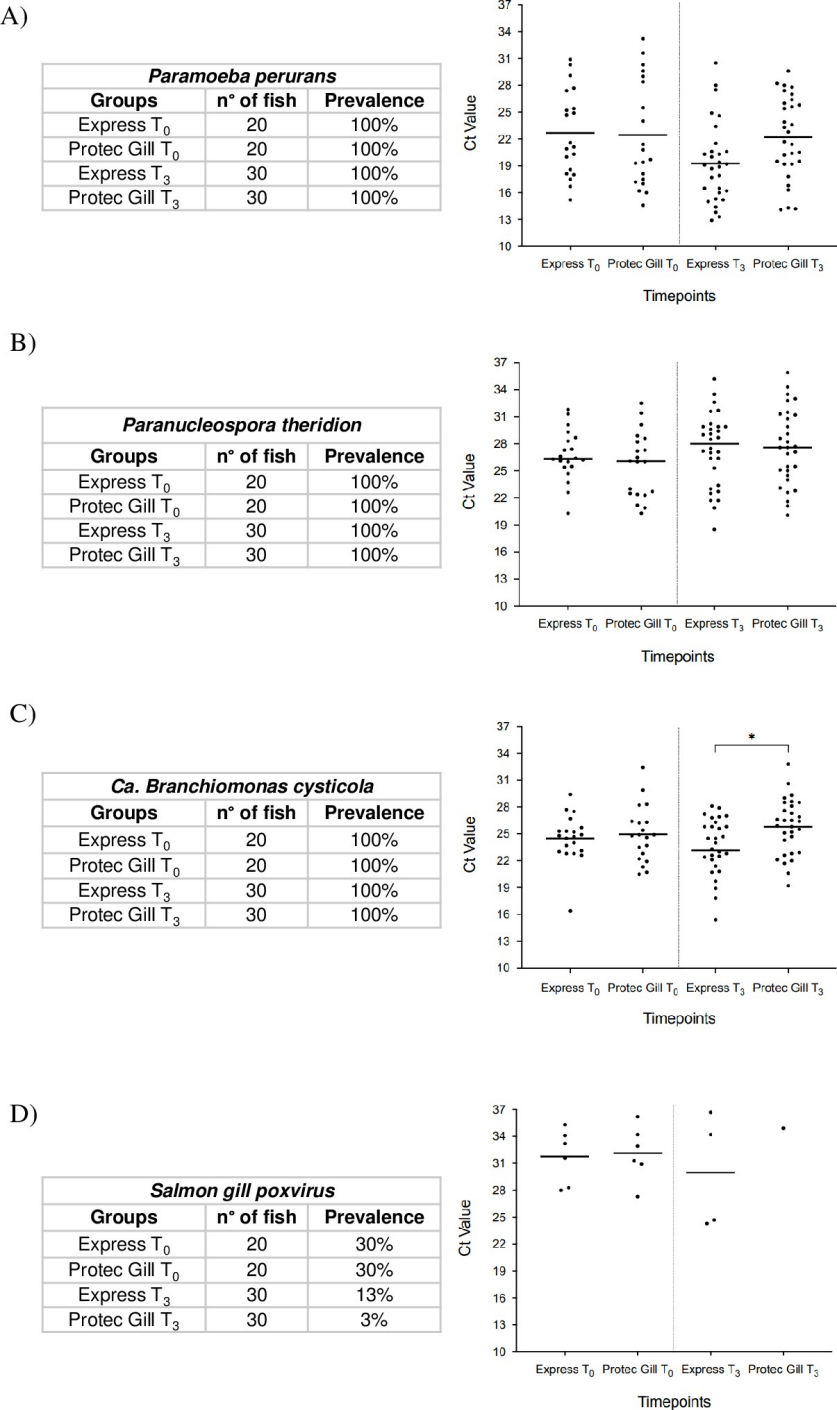
Pathogen detection. Detection of *Paramoeba perurans* (A), *Paranucleospora theridion* (B), *Ca*. *Branchiomonas cysticola* (C), and salmon gill poxvirus (D), by qPCR, at the beginning of the study (T_0_) and at the end (T_3_) in Atlantic salmon gill tissue. Results are reported as mean and individual values; n = 20 (T_0_), n = 30 (T_3_). Ct value = cycles to threshold value, * p < 0.05.

A significant decrease in pathogen load (higher Ct value) was recorded for *Ca*. *B*. *cysticola* in the Protec Gill group at T_3_ compared to the Express group at the same timepoint (Fig 5). No statistical differences were recorded for *P*. *perurans* and *P*. *theridion* detection when comparing the two groups. As for SGPV presence, in the Protec Gill group at T_3_ there was a lower number of fish in which the virus was detected (3% prevalence, only one fish with high Ct value), compared to the Express group at the same timepoint (13% prevalence). Since SGPV was detected in only one fish in the Protec Gill group, no statistical analysis was carried out.

#### Gill histology

**Fig 6:**
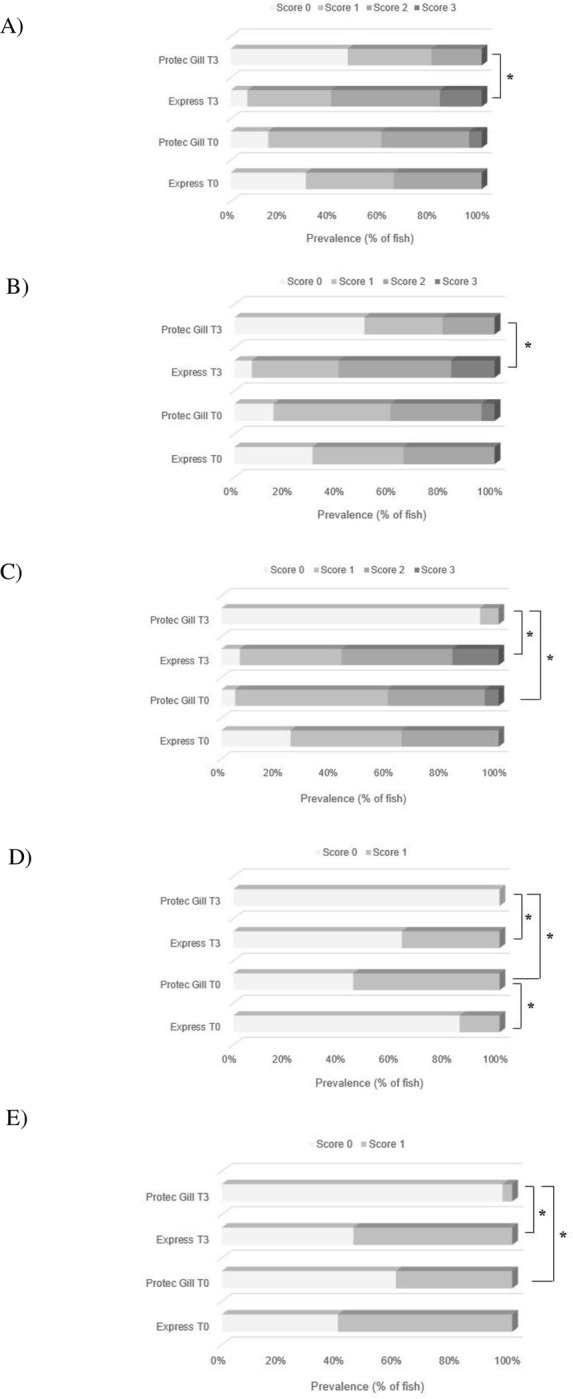
Gill histology. Histopathological analysis of gill lesions analysed in Atlantic salmon gill tissue at the beginning of the study (T_0_) and at the end (T_3_): (A) epithelial and mucous hyperplasia, (B) lamellar fusion, (C) tissue degeneration/necrosis, (D) oedema, (E) pathogen load. Results are reported as prevalence percentage of fish with a specific score (from 0 to 3); n = 20 (T_0_), n = 30 (T_3_), * p < 0.05.

There was a significant reduction in all the analysed gill lesions (epithelial and mucous hyperplasia, lamellar fusion, necrosis, oedema, and pathogen load) in the Protec Gill group at T_3_ compared to the Express group (Fig 6). For epithelial and mucous hyperplasia and lamellar fusion, 50% of the investigated fish population had no lesions (score 0) in the Protec Gill group at T_3_ compared to the Express group (Fig 6A and 6B). A significantly lower number of fish (7%) had mild necrotic changes (score 1) at T_3_ in the Protec Gill group compared to the Express group, which had lesions of all three severities (score 1 to 3) (Fig 6C). Interestingly, Protec Gill group showed less necrosis at T_3_ compared to T_0_, which showed necrosis of all three severities (score 1 to 3). Moreover, at T_3_ there was no evidence of oedema or fluid accumulation in the gill tissue of fish in the Protec Gill group compared to the Express group (Fig 6D). The pathogen load, defined as presence of bacteria (epitheliocystis) and parasites (amoebae) in gills, decreased significantly in the Protec Gill group at T_3_ compared to the Express group at the same timepoint, and compared to the Protec Gill group at T_0_ (Fig 6E).

#### Plasma parameters

**Fig 7:**
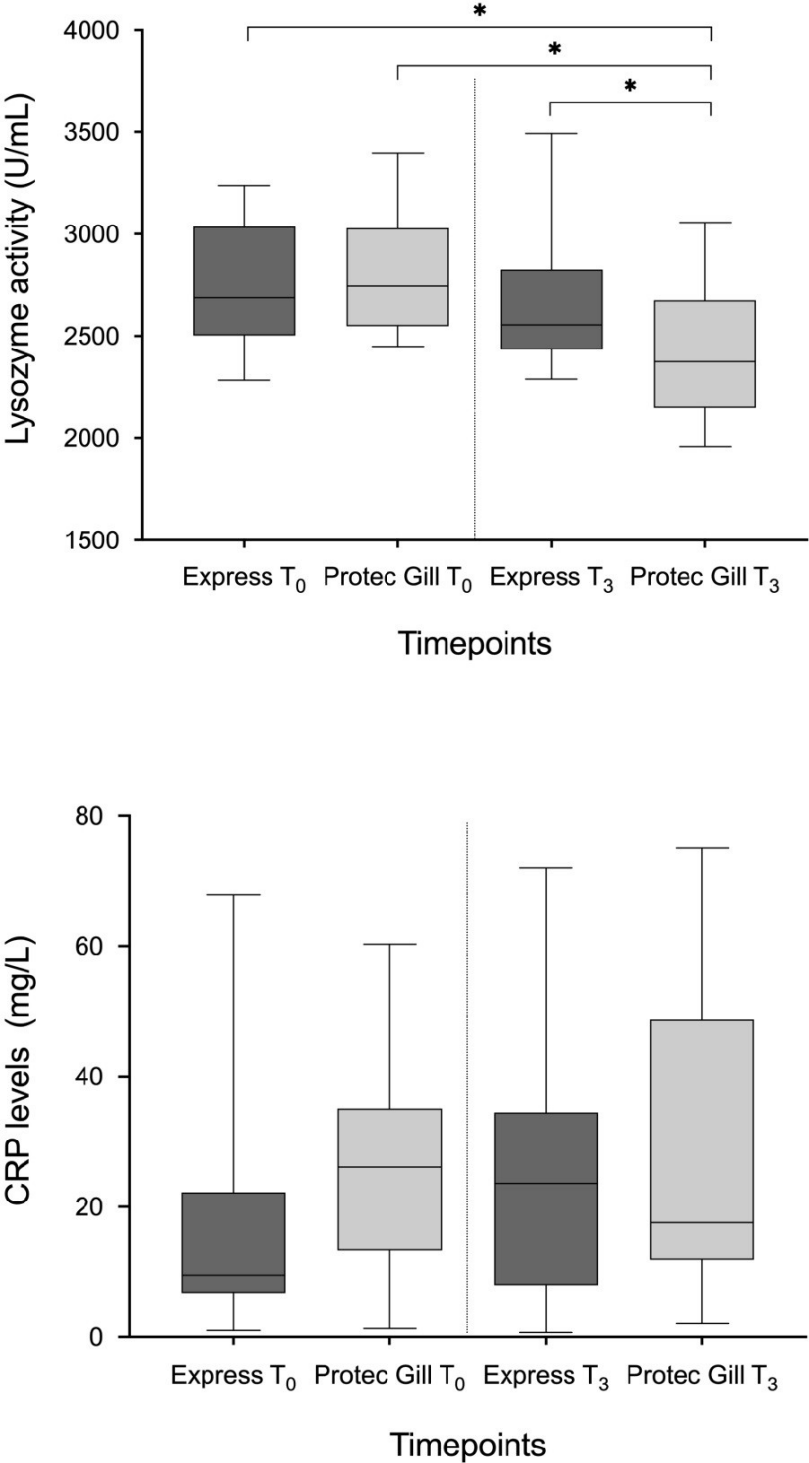
Plasma parameters. Lysozyme (A) expressed U/mL, and C-reactive protein (CRP) (B) expressed in mg/L, activity in Atlantic salmon plasma at the beginning of the study (T_0_) and at the end (T_3_). Results are reported as median ± standard deviation; n = 20 (T_0_), n = 30 (T_3_), * p < 0.05.

A significant decrease in the lysozyme activity was observed in the Protec Gill group at T_3_ compared to the Express group at the same timepoint and compared to both groups at T_0_ (Fig 7). No statistical differences were recorded in the CRP values between groups at any timepoint.

## Discussion

*In vitro* cell cultures are an important part of modern research, as they represent a good alternative to *in vivo* testing [38]. Cell lines derived from fish is a promising tool for studying many of the aquaculture challenges such as fish growth, disease assessment and reproduction [39–41]. They also can be effectively used as model systems to investigate nutrient assimilation and metabolism, but rarely have been used to study aspects of fish nutrition [39]. Most of the *in vitro* studies on fish nutrition are using the intestinal rainbow trout epithelial cell line (RTgutGC) [42–44], for understanding the functional immunity system of the fish gut as well as the effects of functional feed ingredients in the gut cells [42]. However, in the case of CGD, the use of gill cells seems to be a more appropriate tool to assess the beneficial effects of functional ingredients. Since both gut and gill fish cell lines from Atlantic salmon are not well established as *in vitro* system and are not commercially available, in the current study the suitability of RTgill-W1 cell line was investigated.

Based on the cell viability assay, thresholds of the ingredients’ concentration were identified. When applied at high concentrations, all tested functional ingredients, except vitamin C, significantly reduced cell viability. A clear dose-response curve was observed, where increasing the concentration decreases the viability, probably associated with a decrease in metabolic activity. Furthermore, these results contributed to the identification of the ideal ingredient concentrations ranges, which might be useful in establishing the concentration to be included in future diets.

Concerning the production of ROS, the effects differed between ingredients. Arginine and β-glucan showed an increase in ROS production, which was significant at 1–100 μg/mL for arginine and between 0.01 and 1000 μg/mL for β-glucan. According to previous study, β-glucan enhance non-specific host defense mechanisms, stimulating leukocytes to trigger their phagocytic reactions, through the production of ROS [10], which can explain the present study finding. Instead, no improvement was reported at cellular level when RTgill-W1 cells were exposed to arginine, used as single ingredient. In fish, antioxidant effects of arginine have been previously evaluated [45–48], but no significant effects were found, in accordance with the current study’s findings. However, arginine beneficial contribution might be higher when included in diet formulation, due to positive interaction with other ingredients. Interestingly, in our study, when amoebae were exposed to arginine, a clear amoebicidal activity at the highest concentrations (10000 and 1000 μg/mL) was observed, highlighting a potential role of arginine as an amoebicidal agent to prevent microbial infections. This feature has not yet been thoroughly explored and further research is needed to understand the mechanism behind this aspect.

Vitamin C, as widely reported [8, 49, 50], mainly exerted antioxidant properties, showing a significant decrease in ROS production at all tested concentrations (0.1 to 5000 μg/mL). According to previous study, this beneficial effect is exerted at low concentration, like 0.1 μg/mL, which could be safely used in future fish feed formulation [49].

Two PFAs were tested for assessing their potential as antioxidant and as amoebicidal agents to prevent microbial infections. The PFA 1 showed a significant increase in ROS production at concentrations higher than 1000 μg/mL, highlighting a potential cytotoxic effect if provided at high dosage. At low concentrations (0.0001 and 0.001 μg/mL) showed instead a significant decrease in ROS production and might be therefore used for its antioxidant properties. In a concentration range of 0.01 to 100 μg/mL, the second phytogenic, PFA 2, significantly reduced the production of ROS, recommending its usage in this range for exerting antioxidant properties. According to a previous study, increasing the level of PFAs is not associated with improvements of growth performance and feed utilization efficiency [51], which can explain the present study finding, where both PFAs exerted antioxidant properties at low dosages. Regarding their potential amoebicidal activity, a significant decrease in the survival of amoebae was observed for both tested PFAs, especially for PFA 2 which also showed a significant decrease at the lowest tested concentration (100 μg/mL). According to previous studies [3, 13], PFAs are recognized to have antibacterial, antiviral, antifungal properties and, sometime, immune stimulatory and antimicrobial effects in fish, in accordance with our findings. Overall, the inclusion of PFAs with antimicrobial activity in dietary supplement appears to be a promising alternative for preventing microbial infections [14, 15].

Currently available methods for preventing and treating fish diseases are expensive and shown limited efficacy [52, 53]. Moreover, as no vaccines nor specific treatments are licensed to treat CGD, the optimization of diet formulation might be a successful strategy to improve fish health and decrease disease susceptibility [17]. Herein, the beneficial effects of a commercially available functional feed, Protec Gill, were investigated in fish affected by CGD.

The most effective, non-invasive, and commonly used means for the assessment of gill diseases on a commercial scale is carried out through the gross pathological evaluation of gill arches to identify focal/multifocal lesions [54, 55], and through the evaluation of growth and mortality rates. Although, these methods might not be sensitive enough to show fish health improvements during feeding experiments, as in the current study. Herein, fish affected by CGD fed with two different diets had similar AGD and PGD scores after three weeks, and neither mortality nor growth showed significant differences between diet groups. However, a clear improvement in gill tissue was observed through the histopathological assessment. The selected histological parameters, epithelial and mucous hyperplasia, lamellar fusion, tissue degeneration/necrosis, oedema, and pathogen load, are known to be the most representative lesions during gill disease outbreaks [55]. A significant improvement in gill tissue after three weeks of Protec Gill feeding was demonstrated, with a significant reduction of all the analysed gill lesions. Interestingly, in the Protec Gill group after three weeks there was a significant reduction of epithelial and mucous hyperplasia (50% of fish had score 0), which is reported to be one of the predominant characteristics in fish affected by CGD [25], and a significant reduction of pathogen load, in particular the presence of epitheliocystis, which is associate with the presence of *Ca*. *B*. *cysticola* [56], one of the major contributors of CGD [23]. The analysis of plasma parameters, especially the lysozyme activity, also confirmed a reduction in the infection of fish after three weeks of Protec Gill feeding. CRP levels were not able to distinguish group conditions after three weeks, probably due to the fact that is more responsive during acute inflammatory episodes [57].

In literature, most of the studies are focused on describing the mechanisms behind the development of CGD, the role of each pathogen involved, and the use of histopathological gill assessment as a more in-depth investigation [23–26, 58, 59]. However, one aspect that has not been yet deeply explored is the investigation of alternative strategies to improve gill tissue conditions and fish health during CGD outbreaks, especially in absence of vaccines or specific treatments. Our study findings suggests that the use of functional diets is a feasible strategy for preventing or mitigating the increasingly common gill diseases, particularly in cases of CGD, and further research could focus on evaluating the use of such functional diets for preventing disease outbreaks.

## Conclusion

In fish, knowledge about basic mode of action of functional ingredients and their interactions when included in a diet is weak and fragmentary. The *in vitro* model, which may be implemented with additional assays and cell line types, appears to be a suitable alternative for screening ingredients, reducing the number of *in vivo* trials for a more sustainable aquaculture practice.

The use of a diet containing selected functional feed ingredients in a field trial was a successful strategy for mitigating the CGD, by slowing disease progression, reducing pathogen load, and significantly improving gill tissue condition.

## Supporting information

S1 File (DOCX)

S1 Appendix Raw data. (XLSX)
