# Correction: Effectiveness of functional feed ingredients to enhance gill disease in Atlantic salmon (*Salmo salar*, L.)

**DOI:** 10.1371/journal.pone.0314952

**Published:** 2024-12-02

**Authors:** The PLOS ONE Staff

## Notice of Republication

An incorrect version of this article was published in error. The publisher apologizes for this error. This article was republished on November 19, 2024, to correct the error. Please download the article again to view the corrected version. Both the originally published, uncorrected article and the republished, corrected article are provided here for reference.

## Supporting information

S1 File Originally published, uncorrected article. (PDF)

S2 File Republished, corrected article. (PDF)
