# Supplementary material for: Effectiveness of functional ingredients to enhance gill disease in Atlantic salmon (Salmo salar, L.)
Source: PLoS One. 2024 Jun 20;19(6):e0304112. doi: 10.1371/journal.pone.0304112 (PMC11189246; doi:10.1371/journal.pone.0304112)
Supplement: S1 File — (DOCX) [file pone.0304112.s001.docx]

**Effectiveness of functional feed ingredients to enhance gill disease in Atlantic salmon (*Salmo salar*, L.)**

**Table 1. Functional feed ingredients.** Functional feed ingredients used for *in vitro* testing. (A) RTgill-W1 assays; (B) Amoebae (*Paramoeba perurans*) survival testing.

(A)

| Ingredient | Supplier | Tested concentration range | Solution preparation |
| --- | --- | --- | --- |
| Arginine | Provided by Skretting | 0.0001 – 2000 μg/mL | 20 mg were dissolved in 10 mL of L-15 media (for cell viability assay) or PBS (for ROS assay), followed by centrifugation at 1000g for 5 min. |
| β-glucan | Provided by Skretting | 0.001 – 1000 μg/mL | 10 mg were dissolved in 10 mL of L-15 media (for cell viability assay) or PBS (for ROS assay), followed by centrifugation at 1000g for 5 min. |
| Vitamin C | Sigma-Aldrich  (A4544-100G) | 0.01 – 500 μg/mL (Cell Viability Assay)  0.1 – 5000 μg/mL (ROS Assay) | 5 mg were dissolved in 10 mL of L-15 media (for cell viability assay) or PBS (for ROS assay). |
| Phytogenic feed additive 1 | Provided by Skretting | 0.0001 – 2000 μg/mL | 20 mg were dissolved in 10 mL of L-15 media (for cell viability assay) or PBS (for ROS assay), followed by centrifugation at 1000g for 5 min. |
| Phytogenic feed additive 2 | Provided by Skretting | 0.0001 – 1000 μg/mL | 10 μL were first dissolved in a low volume of DMSO (0.01%) and then dissolved in 10 mL of L-15 media (for cell viability assay) or PBS (for ROS assay). |

| Ingredient | Tested concentration range | Solution preparation |
| --- | --- | --- |
| Arginine | 10 – 10.000 μg/mL | Dissolved in seawater at 1%, left to soak for 16h and then filtered, using a 0.5 μm nylon filter. |
| Phytogenic feed additive 1 | 10 – 10.000 μg/mL | Dissolved in seawater at 1%, left to soak for 16h and then filtered, using a 0.5 μm nylon filter. |
| Phytogenic feed additive 2 | 10 – 10.000 μg/mL | First dissolved 1:1 in 100% ethanol and then further diluted in seawater to reach the desired concentration. This resulted in traces of ethanol (0.001-1%) being present in the final concentrations. |

(B)

**Table 2.** **Field trial.** Estimated average fish weight (Kg) and number of fish in each cage at the beginning of the study (T_0_).

| **Cage** | **Diet group** | **Average weight at T_0_ (Kg)** | **Number of fish at T_0_** |
| --- | --- | --- | --- |
| 1 | Protec Gill | 3.05 | 145591 |
| 2 | Protec Gill | 2.92 | 152644 |
| 3 | Express 2500 | 3.12 | 154650 |
| 4 | Express 2500 | 3.02 | 152505 |

**Table 3.** **Diet formulations.** Formulation and proximate composition of the feeds used in the field trial.

| **Ingredients** | **Content as % in the Express 2500 feed** | **Content as % in the Protec Gill feed** |
| --- | --- | --- |
| Soy protein concentrate | 21,41 | 21,38 |
| Rapseed oil | 17,64 | 18,66 |
| Wheat gluten | 9,3 | 3,78 |
| Wheat | 8,45 | 7,00 |
| Fishoil crude North-Atlantic | 8,47 | 7,58 |
| Fishoil crude South-American | 3,00 | 3,1 |
| Sunflower meal | 7,00 | - |
| Guar meal | - | 6,8 |
| Fishmeal | 4,33 | 6,79 |
| Pea protein | - | 4,02 |
| Linseed oil | 3,33 | 3,35 |
| Horse beans dehulled | 3,00 | 5,12 |
| Water | 1,70 | 1,96 |
| Rapseed Lecithin | 1,00 | 1,00 |
| Pigment Astaxanthin 10% | 0,07 | 0,07 |
| Mineral Mix | 1,64 | 1,77 |
| Vitamin E Ads 50 % | 0,05 | 0,08 |
| Vitamin C monophosphate 35% | 0,04 | 0,19 |
| L-arginine | - | 0,5 |
| β-glucan | - | 0,1 |
| PFAs | - | 0,5 |

**Table 4.** **Delousing events.** Overview of delousing events of the different cages before and under the trial period. Period numbers indicate weeks before and during the trial.

| **Period** | **Cage 1** | **Cage 2** | **Cage 3** | **Cage 4** | **Method** |
| --- | --- | --- | --- | --- | --- |
| T_-16_ | 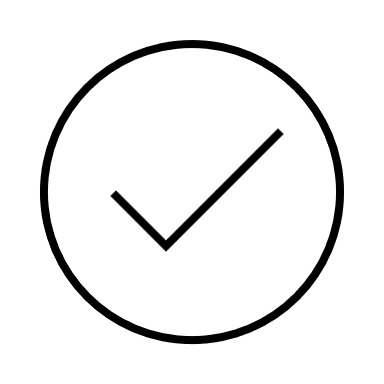 | 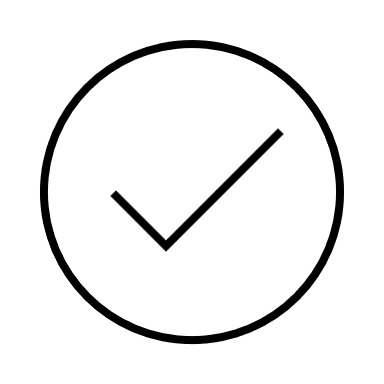 | - | 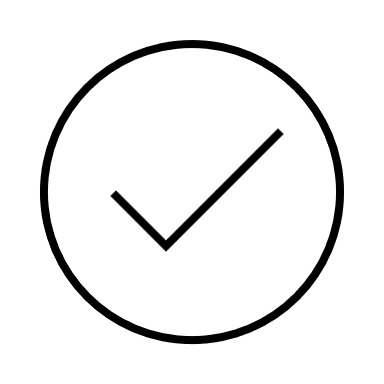 | Thermolicer |
| T_-12_ | 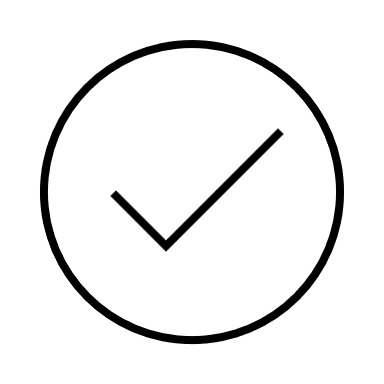 | 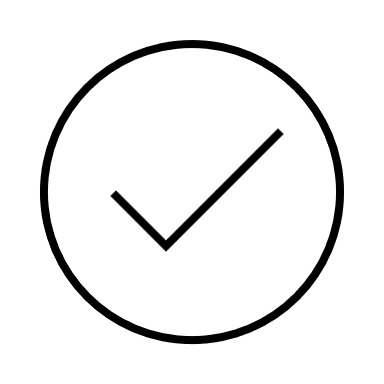 | 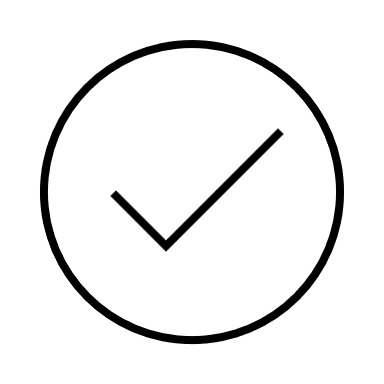 | 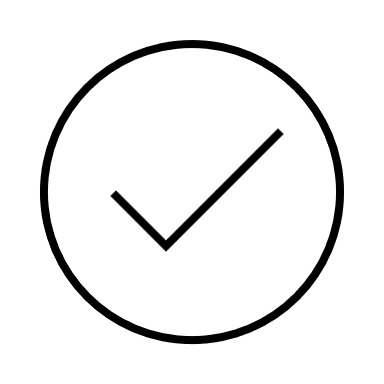 | Thermolicer |
| T_-8_ | 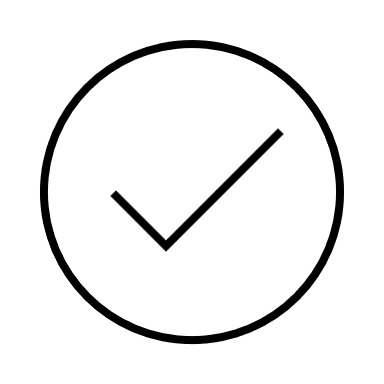 | 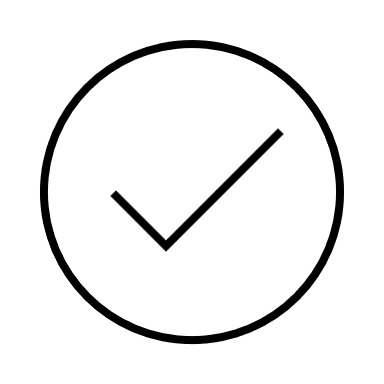 | 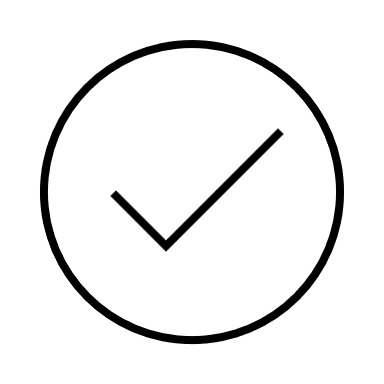 | 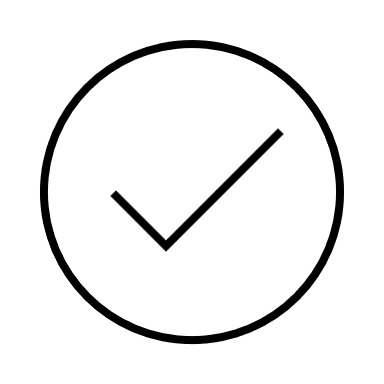 | Thermolicer |
| T_-7_ | 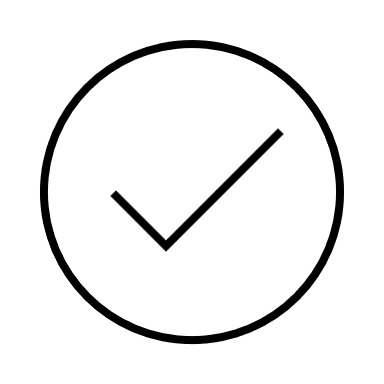 | - | 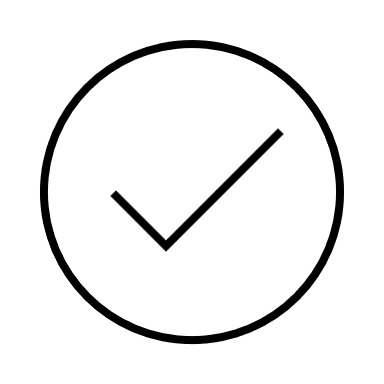 | 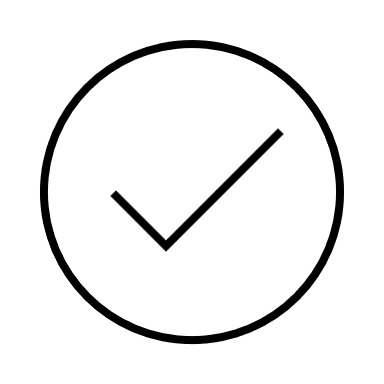 | Optilice |
| T_-4_ | 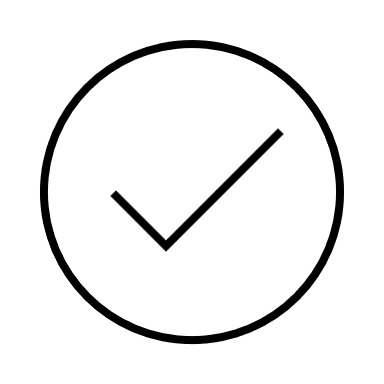 | 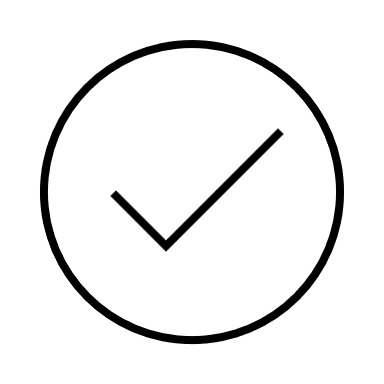 | - | - | Thermolicer |
| T_-2_ | - | - | 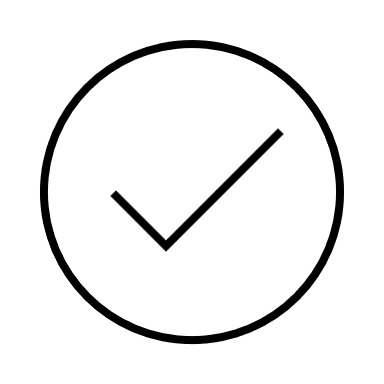 | - | Thermolicer |
| T_-1_ | 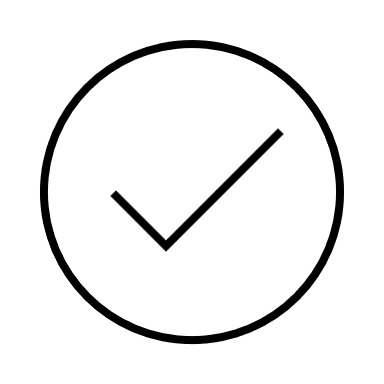 | 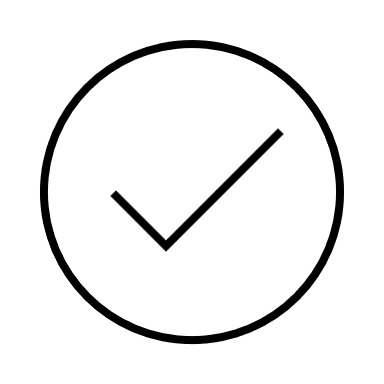 | - | 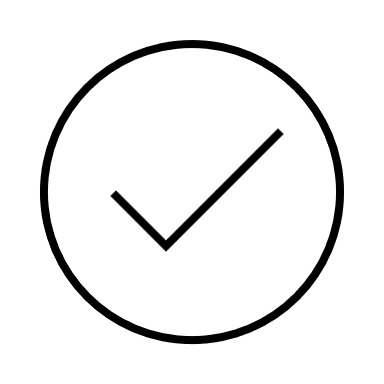 | Thermolicer |
| T_2_ | - | 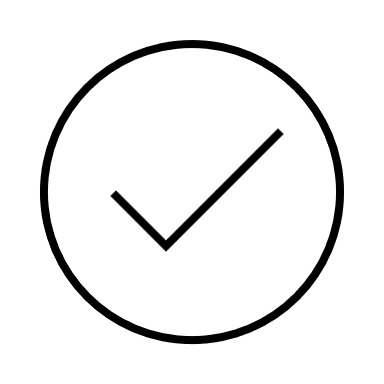 | 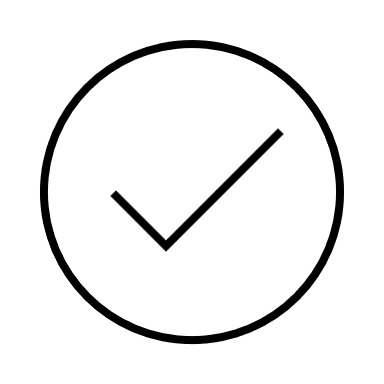 | - | Thermolicer |

**Table 5.** **Histopathological gill score.** Scoring system of the histopathological gill lesions, adapted from [36].

| Score | Epithelial and Mucous Hyperplasia | Lamellar fusion | Tissue degeneration/Necrosis | Oedema | Pathogen Load  (Epitheliocystis) | Pathogen Load  (*Neoparamoeba* spp.) |
| --- | --- | --- | --- | --- | --- | --- |
| 0 | None or very minor | None or very minor | None or very minor | Absence -Background lamellar oedema <10 % of gill tissue affected | Absence | Absence |
| 1 | Mild increase in lamellar epithelial cell (< 10% of gill tissue affected) | Occasional focal fusion of filaments (<10 % of gill tissue affected) | Scattered, occasional, degenerating necrotic or apoptotic cells (< 10% of gill tissue affected | Presence - lamellar oedema above 10 % of gill tissue affected | Presence | Presence |
| 2 | Moderate multifocal or widespread increase in lamellar epithelial cells, affecting 10-50 % of the tissue | Multifocal areas of fusion, affecting 10-50 % of gill tissue | Multifocal, degenerating necrotic or apoptotic cells affecting 10-50% of the tissue | - | - | - |
| 3 | Extensive multifocal or widespread increase in lamellar epithelial cells, affecting > 50% of the tissue | Extensive fusion and loss of normal architecture, affecting > 50 % of the tissue | Extensive, degenerating necrotic or apoptotic cells, affecting > 50 % of the tissue | - | - | - |


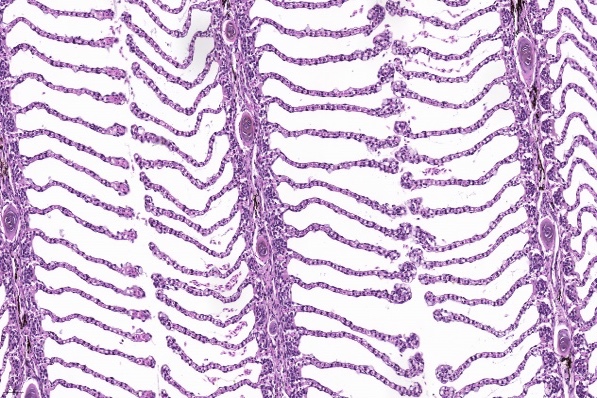

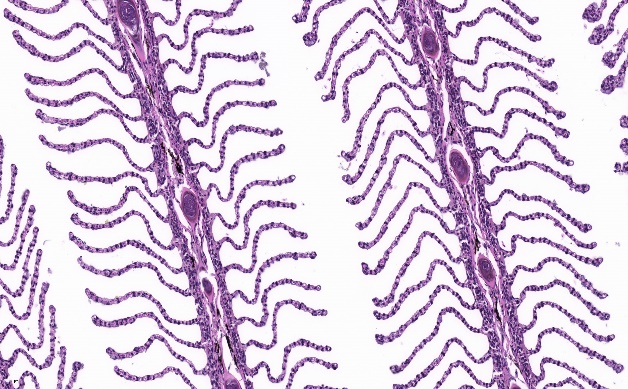


**A)**

**B)**

**SCORE 0**


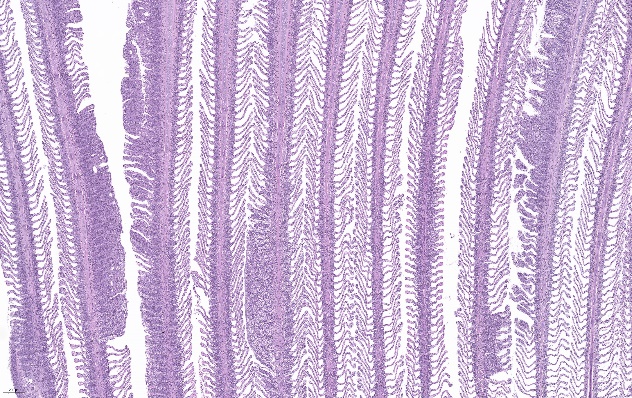

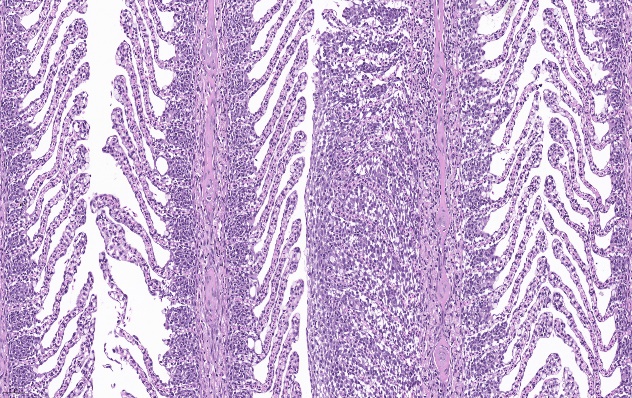


*

**C)**

**D)**

**SCORE 1**


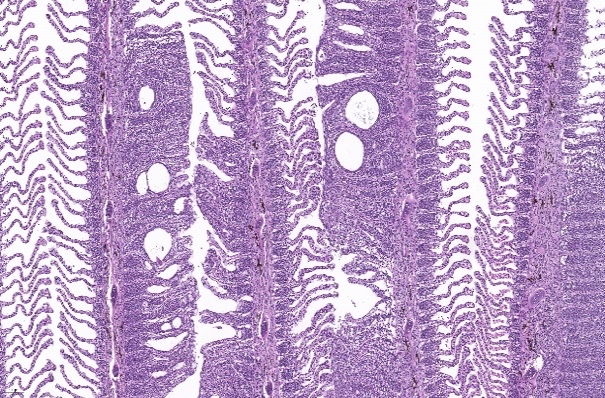


*


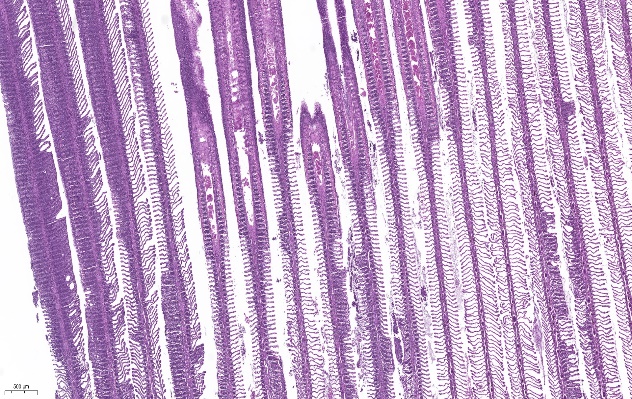


**E)**

**F)**

**SCORE 2**


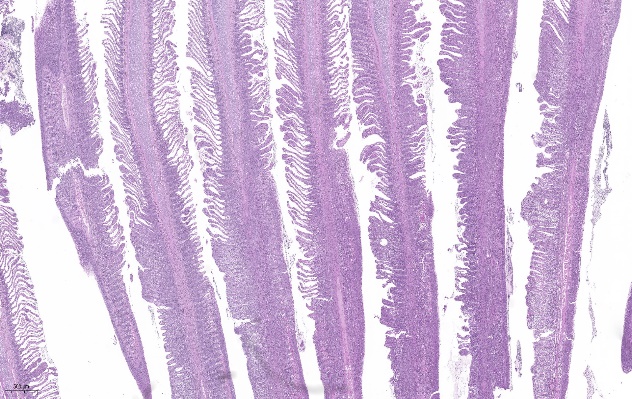

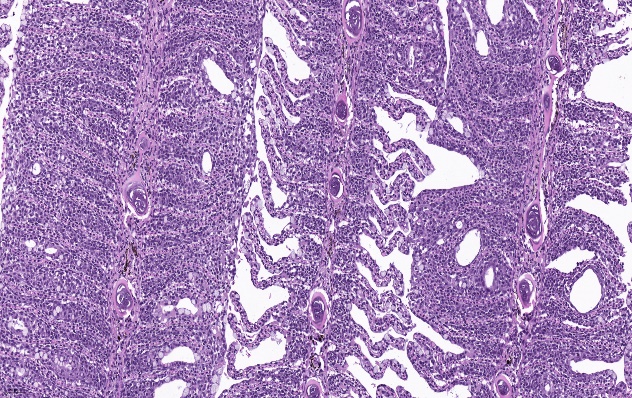


**G)**

**H)**

**SCORE 3**

**Fig 1.** **Images scoring system.** Representative images of the semiquantitative scoring system described in S5 Table, gill histopathology as low (A, C, E and G) and high (B, D, F, and H) power view. Score 0: none to focal lesions such as clubbing (arrow) and fusion. Score 1: (C) mild multifocal areas of lesions (arrows) comprise of epithelial hyperplasia, fusion, and inflammation (D, star). Score 2: (E) moderate multifocal patches of lesions (arrow) and (F) vesicle formation. Score 3: (G) severe widespread increase in lamellar hyperplasia/fusion and (H) inflammation (arrow). Reprinted from Dr. Naveed M. Yousaf under a CC BY license, with permission from PLOS ONE, original copyright 2024.


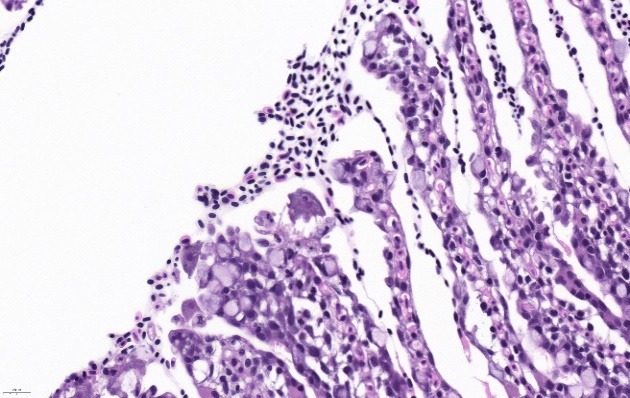


**A)**


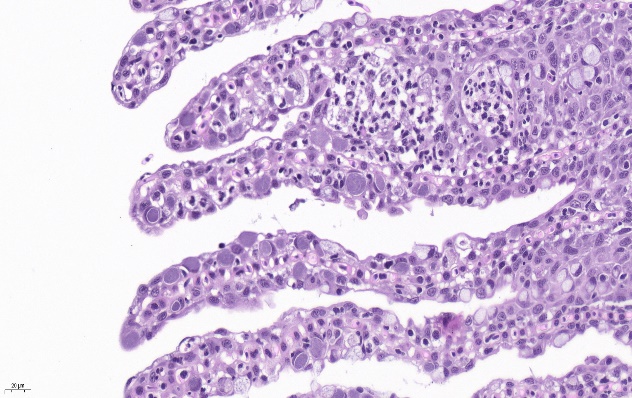


**B)**

*


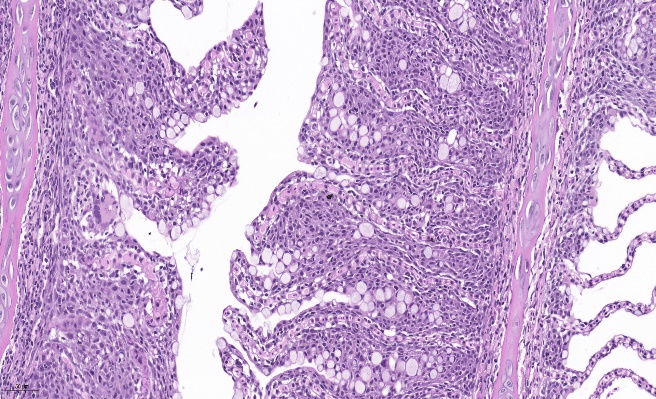


**C)**


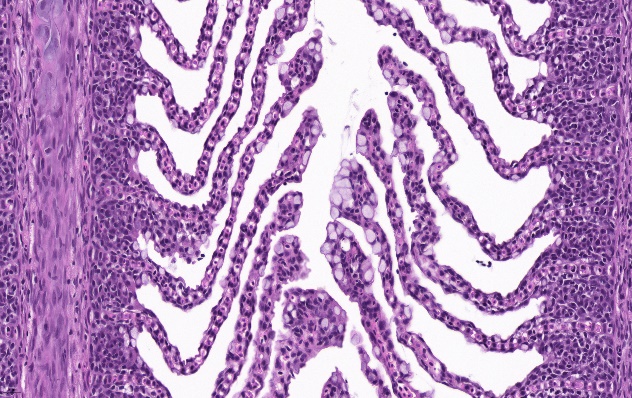


**D)**

*


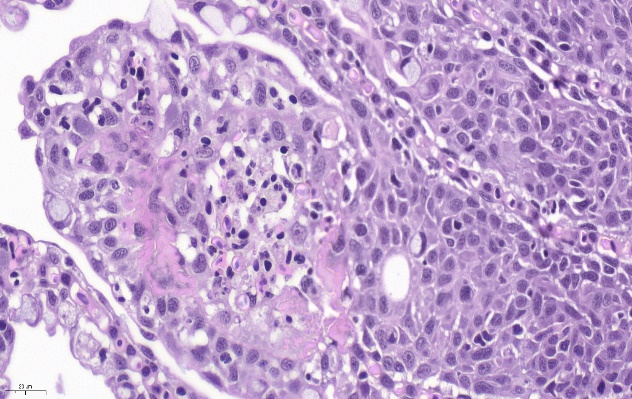


*

**E)**

**Fig 2.** **Images gill lesions.** Histological sections of gills from Atlantic salmon (*Salmo salar*) stained with hematoxylin and eosin. Representative image showing: (A) amoeba (arrow) surrounding the secondary lamella; (B) multiple micro-cysts (epitheliocystis, arrow) in the epithelial cells as regular round to oval could be seen in the secondary lamella; (C) epithelial hyperplasia and fusion of secondary lamella with inflammation (star) were noted; (D) epithelial and mucous hyperplasia with fusion could been seen; (E) focal areas of necrosis with vacuolization (star). Reprinted from Dr. Naveed M. Yousaf under a CC BY license, with permission from PLOS ONE, original copyright 2024.

**
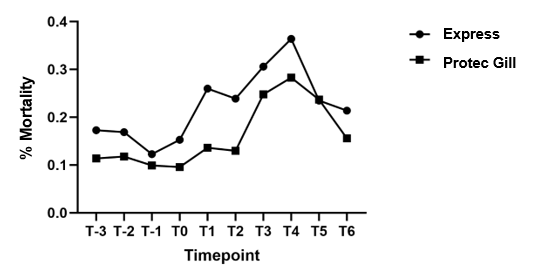
Fig 3. Mortality.** Weekly mortality relative to the fish count in each group. Timepoints are reported in weeks starting from three weeks prior to the trial (T_-3_) until three weeks post-trial (T_6_).
