# Supplementary material for: Correction: Effectiveness of functional feed ingredients to enhance gill disease in Atlantic salmon (Salmo salar, L.)
Source: PLoS One. 2024 Dec 2;19(12):e0314952. doi: 10.1371/journal.pone.0314952 (PMC11611145; doi:10.1371/journal.pone.0314952)
Supplement: S2 File — (PDF) [file pone.0314952.s002.pdf]

RESEARCH ARTICLE

Effectiveness of functional feed ingredients to enhance gill disease in Atlantic salmon (*Salmo salar*, L.)

Matteo Vitale[1,2]*, **Eirik Hoel**[3], **Muhammad Naveed Yousaf**[2], **Martha Amalie Kambestad**[4], **Julia Mullins**[2], **Leidy Lagos**[2], **Kjetil Berge**[3], **Charles McGurk**[2], **Daniela Maria Pampanin**[1]

1 Department of Chemistry, Bioscience and Environmental Engineering, University of Stavanger, Stavanger, Norway, **2** Skretting Aquaculture Innovation, Stavanger, Norway, **3** Skretting AS, Stavanger, Norway, **4** Bremnes Seashore, Bremnes, Norway

* matteo.vitale@uis.no

Check for updates

OPEN ACCESS

Citation: Vitale M, Hoel E, Yousaf MN, Kambestad MA, Mullins J, Lagos L, et al. (2024) Effectiveness of functional feed ingredients to enhance gill disease in Atlantic salmon (*Salmo salar*, L.). PLoS ONE 19(6): e0304112. https://doi.org/10.1371/journal.pone.0304112

Editor: Amel Mohamed El Asely, Benha University, EGYPT

Received: November 24, 2023

Accepted: May 6, 2024

Published: June 20, 2024

Copyright: © 2024 Vitale et al. This is an open access article distributed under the terms of the Creative Commons Attribution License, which permits unrestricted use, distribution, and reproduction in any medium, provided the original author and source are credited.

Data Availability Statement: All relevant data are within the paper and its Supporting Information files.

Funding: Research Council of Norway, grant #336672 In vitro methods for screening novel ingredients, for the funding under the Innovation Project Industrial Sector Ph.D. program.

Competing interests: The authors have declared that no competing interests exist.

Abstract

Herein, the feasibility of *in vitro* methods for screening the qualities of functional feed ingredients using the fish cell line RTgill-W1 and the culture of *Paramoeba perurans* was investigated. Five functional ingredients (arginine, β-glucan, vitamin C, and two phytogenic feed additives) were selected to investigate their effects on cell viability and reactive oxygen species production. Three of them (arginine and two phytogenic feed additives) were additionally tested to assess their potential amoebicidal activity. As these functional ingredients are the core of a commercial feed (Protec Gill, Skretting AS), beneficial effects were further assessed in a field trial using Atlantic salmon (*Salmo salar*), affected by complex gill disease (CGD).

Threshold levels of the ingredient concentrations were identified through RTgill-W1 cell viability assay. High concentrations of all tested ingredients, except vitamin C, resulted in a significant reduction of cell viability. Different effects on reactive oxygen species production were observed among the ingredients. Arginine and two phytogenic feed additives exhibited also pronounced amoebicidal activity at the highest tested concentrations.

In the field study, three weeks of feeding Protec Gill slowed the disease progression, and also led to a significant improvement in gill tissue conditions. Significant reductions of epithelial and mucous hyperplasia, predominant symptoms in fish affected by CGD, and pathogen load, particularly epitheliocystis associated with *Ca*. *Branchiomonas cysticola*, one of the major contributors of CGD, were observed. These positive outcomes underscore the potential of functional feed ingredients in ameliorating gill diseases and enhance fish health.

RTgill-W1 cell line assays proved to be effective tools for screening functional ingredients, providing valuable insights into optimal concentration ranges. Implementing an *in vitro* cell-based approach, with additional assays and cell line types, it's a promising alternative to reduce the number of animals used during fish trials, for a more sustainable aquaculture practice.

Introduction

In the last decades the expansion of the aquaculture industry has been accompanied by a significant rise in fish feed production, which has led increased pressure on marine supplies such as fish meal and fish oil, resulted in low availability and high prices [1]. Reducing reliance on marine commodities by extending the use of alternative ingredients will improve the future sustainability of the aquaculture practice. The application of functional feed ingredients represents a great opportunity to advance fish growth and health, boost the immune system, and induce physiological benefits beyond those provided by traditional feed [2]. Numerous dietary supplements with immunomodulatory properties can be used to stimulate the immune system of fish [3–4], such as single amino acids like arginine [5–6], vitamins [7–8], β-glucans [9–11], and phytogenic feed additives (PFAs) [12–15], which use has been recently introduced in aquaculture. Functional feed ingredients may also play an important role in lowering disease susceptibility [16–17].

In European salmon-producing countries like Norway, Scotland and Ireland, gill diseases have become one of the most significant health challenges for the aquaculture industry [18–20]. They can be classified as either simple or complex/multifactorial gill disease, based on a presumption of single or multiple causes and infectious agents involved in the pathogenesis [21–22]. Typically, the term multifactorial gill disease or complex gill disease (CGD) is used to describe the type of gill illness in which multiple causes can be observed simultaneously and there are no obvious primary causal agents [21, 23–26]. CGD encompasses syndromes referred to as proliferative gill inflammation (PGI) and proliferative gill disease (PGD) [26]. PGI is a pathology-based diagnosis first described in Norway, in which gills mainly present a combination of lamellar vascular changes, inflammation, cell death, epithelial and mucous hyperplasia [27]. PGD has been used as a non-specific term for the examination of gross lesions in the salmon gills [26], and as a general descriptive term for proliferative changes in the gill epithelium [28].

The aim of the present study was to evaluate the feasibility of *in vitro* methods for screening the qualities of functional feed ingredients, using the fish cell line RTgill-W1, which has never been used to study aspects of fish nutrition, and the culture of *Paramoeba perurans*. As the selected functional ingredients are the core of a commercial feed (Protec Gill, Skretting AS), their beneficial effects were further assessed in a field trial using Atlantic salmon (*Salmo salar*), affected by CGD.

Materials and Methods

Functional feed ingredients

Five functional feed ingredients were used in this study: arginine, β-glucan, vitamin C, and two PFAs. β-glucan and PFAs are proprietary composition of Skretting ARC and encompassing confidential information. All stock solutions were freshly prepared and used immediately for the assays (S1 Table).

RTgill-W1 cell culture

RTgill-W1 cells (American Type Culture Collection No. CRL-2523, commercially available), obtained from gill explants of adult rainbow trout (*Oncorhynchus mykiss*), were cultured using 75 cm^2 tissue culture flasks (Corning Inc., NY, USA) (19˚C, w/o CO_2, in the dark), in Leibovitz's (L-15) media (Gibco, NY, USA), containing 10% (v/v) of fetal bovine serum (Biowest, Nuaillé, France), 100 U/mL penicillin and 100 μg/mL streptomycin (Gibco, NY, USA) [29]. Confluent cells were exposed in 96-well plates (3×10^4 cells per well) to a range of

concentrations of the selected functional feed ingredients (S1A Table). For each concentration seven replicates were tested (n = 7).

Cell viability assay

Cell viability was assessed using a fluorometric assay as described by [30], with small adjustments. Confluent cells were exposed for 24 h to each ingredient. Control (cells with only L-15), blank (only L-15) and positive control (hydrogen peroxide, H_2O_2, 100 μM, diluted in L-15) were also tested. After incubation, the exposure solution was removed and the resazurin dye (484 μM, AlamarBlueTM, ThermoFisher Scientific) was added (1:10 ratio with L-15). The ratio of viable cells was quantified using a microplate reader (SpectraMax Paradigm Multi-Mode), which measured the fluorescence of resorufin at excitation and emission wavelengths of 530 and 590 nm, respectively. Relative fluorescence units (RFUs) were normalized to the control.

Reactive oxygen species production assay

Reactive oxygen species (ROS) production was measured by applying a modified version of the protocol described by [31]. Once the cell monolayer was formed, the media was carefully removed, followed by a wash with phosphate buffer saline (PBS). The 2',7'- dichorodihydrofluorescein diacetate (H2DCF-DA) (ThermoFisher Scientific, MA, USA) probe was used as ROS production indicator. It was freshly prepared and 100 μL of 10 mM solution were added in each well (except for the blank), following an incubation time of 30 min. Afterwards, the probe was removed and two additional washes with PBS were done. Then, RTgill-W1 cells were exposed for 60 min to each ingredient. Control (cells with only PBS), blank (only PBS) and positive control (H_2O_2, 100 μM, diluted in PBS) were also tested. The fluorescence emitted due to the oxidation of H2DCF-DA was read using a microplate reader (SpectraMax Paradigm Multi-Mode) at excitation and emission wavelengths of 485 and 528 nm, respectively. RFUs were normalized to the control.

Amoebae survival *in vitro* testing

The culture of *Paramoeba perurans* was obtained from ILAB in cell culture flasks containing malt yeast broth (MYB; malt and yeast extract in 75% seawater). The amoebae were kept in an incubator at 15˚C and sub-cultured in MYB weekly or biweekly, depending on the density of the amoebae and the density of co-occurring bacteria in the cultures, which the amoebae depend upon [32]. Prior to the test, amoebae attached to the flask or floating were concentrated by a centrifugation step at 1000g for 15 min before being resuspended in 5 mL aseptic seawater (ASW). Pelleted amoebae were then mixed with an ingredient (S1B Table) in a 24 cell-well plate. Amoebae culture (1×10^5 per well) were exposed for 24 h to each ingredient in seawater, in addition to the control of seawater only. Then amoebae were pelleted at 1×10^4 cells and resuspended in 200 μL of seawater containing 10 μg/mL fluorescein diacetate (3',6'-diacetylfluorescein) (ThermoFisher Scientific, Prague, Czech Republic; live cells stain) and with 1 μg/mL propidium iodine (2-[4-(2-hydroxyethil)-1-piperazinyl] ethanesulfonic acid) (ThermoFisher Scientific, Prague, Czech Republic; dead cells stain) in the dark, at room temperature, for 10 min. Live and dead cells were quantified using a flow cytometer (BD FACSCanto II, BD Biosciences).

Field trial

The field feeding trial was conducted in a commercial farming site on the west coast of Norway (Bremnes, Norway), where the functional diet Protec Gill (Skretting AS) was tested in

comparison with a standard high performance commercial feed (Express 2500, Skretting AS). The trial started at the end of September 2020 and was conducted over a period of three weeks. Fish (Atlantic salmon, *Salmo salar*) were initially divided into four cages (S2 Table). For the whole experimental period (from T_0 to T_3), two cages (1 and 2) were fed Protec Gill, whereas the two other cages (3 and 4) received Express 2500. An air-pressure driven central feeding system was used for delivering feed to each cage through polyethylene-hoses. All feed used (9 mm diameter) were formulated and produced by extruding technology according to standard procedures in a commercial fish factory (Skretting, Stavanger, Norway) (S3 Table). One month prior to the trial, gills from ten fish were subjected to histopathological analysis by Fish Vet Group AS (Oslo, Norway), providing findings compatible with CGD, through evidence of epithelial and mucous hyperplasia, hyperplastic multifocal gill inflammation, necrotic/apoptotic epithelial cells, presence of epitheliocystis and amoebae. In addition, the presence of pathogens such as *Paramoeba perurans*, *Ca. Branchiomonas cysticola*, *Paranucleospora theridion* (syn. *Desmozoon lepeophtherii*), and salmon gill poxvirus (SGPV) in the gill tissue were confirmed by qPCR analysis.

Field trial design and husbandry

Prior to the trial, all cages were deloused 4–6 times using tempered seawater with Thermolicer or Optilice technology (S4 Table). Only cage 2 and 3 were treated using Thermolicer during the trial period (at T_2). Four-five days before delousing, fish did not receive feed. All pens were cleaned using high pressure seawater according to standard procedures, approximately once a week, before and during the trial period.

Feeding and mortality were registered in a production management system (Mercatus Farmer, Scale Aquaculture AS, Norway). Dead fish were registered and removed from each cage daily.

The weight of the fish was estimated using a production management system based on initial number of fish and weight, feeding and mortality. Specific growth rate (SGR) was calculated based on data from AKVA Fishtalk production management using the following equation:

$$\text{SGR}\ (\%\ \text{growth per day}) = \left(\left(\frac{FBW}{IBW}\right)^{\frac{1}{days}} - 1\right) x\ 100$$

where FBW is the final body weight and IBW is the initial body weight of fish.

This research was carried out in strict accordance with Norwegian aquaculture production and animal welfare regulations (Forskrift om drift av akvakulturanlegg 34. Avlivning av fisk). No approval was necessary as commercial farm samplings in the field do not need to be approved by the Norwegian Food Safety Authority (FOTS),. A permit for the field site access was not required since it was a commercial production site, and all testing were done on fish post-mortem.

Fish were randomly caught using a large seine and euthanized with an overdose of benzocaine (Benzoak vet., ACD Pharmaceuticals AS, Leknes, Norway), with every effort made to reduce the pain.

Macroscopic score of gills

Amoebic gill disease (AGD) scoring was calculated according to [33]: score 0 (clear), no sign of infection and healthy red colour; score 1 (very light), one white spot, light scarring or undefined necrotic; score 2 (light), 2–3 spots and small mucous patch; score 3 (moderate),

established thickened mucous patch or spot grouping up to 20% of gill area; score 4 (advanced), established lesions covering up to 50% of gill area; score 5 (heavy), extensive lesions covering most of the gill surface.

The PGD was scored according to a system developed by Mowi Scotland. Score 0: normal gills and no pathological changes; score 1: very slight thickening or very few lamellae affected; score 2: frequent thickening, but tips only; score 3: almost all lamellae have thickened tips, and some have thickenings progressing to 50% of the length of the lamellae; score 4: most lamellae have thickenings progressing to more than 50% of the length of the lamellae; score 5: almost all lamellae are thickened along entire length.

Fish were analysed per diet group for each sampling point (T_0, n = 20; T_3, n = 40).

Pathogen detection

Gill tissue (2 x 2 x 5 mm) from the middle of the second arch was placed in RNA-later and shipped on ice to Patogen AS (Ålesund, Norway) to perform qPCR analysis of targeting *P. perurans*, *Ca. B. cysticola*, *P. theridion* and SGPV. The qPCR analysis was validated to ISO17025 standards by Patogen AS. Samples were defined as positive when having cycles to threshold (Ct) value lower than 37.0. Elongation factor 1α (EF1α) served as an internal reference gene for all qPCR analysis performed [34]. Results were expressed in Ct of PCR, a relative value that represents the cycle number at which the amount of amplified DNA reaches the threshold level. Low pathogen load was represented by high Ct value.

Fish were analysed per diet group for each sampling point (T_0, n = 20; T_3, n = 30).

Gill histology

Gill tissue from the second gill arch was fixed in 10% neutral phosphate buffered formalin (VWR, International AS, Oslo, Norway) for histopathological evaluation. Samples were processed, sectioned at 2 μm and stained with hematoxylin and eosin [35]. Sections were evaluated for tissue changes using a light microscope according to a scoring system adapted from [36] (S5 Table). The analysed gill lesions included: epithelial and mucous hyperplasia, lamellar fusion, and tissue degeneration/necrosis, all scored from 0 (none or very minor lesions) to 3 (extensive multifocal lesions). In addition, the absence (0) or presence (1) of oedema and pathogen load were recorded.

Representative images of the scoring system are reported in S1A–S1H Fig. Pathogen such as amoeba (S2A Fig) and cysts such as epitheliocystis (S2B Fig), epithelial hyperplasia and lamellar fusion (S2C Fig), epithelial and mucous hyperplasia (S2D Fig) and necrosis (S2E Fig) were identified. The slides were digitized using a slide scanner (Panoramic SCAN II, 3 Dhishtech) for images.

Fish were analysed per diet group for each sampling point (T_0, n = 20; T_3, n = 30).

Plasma parameters

Immediately after catching, blood samples were taken from fish tail vessels (vena/arteria caudalis) by Vacuette containers containing lithium-heparin. Samples were centrifuges immediately after collection at 4000 g for 6 min. Then the plasma was frozen and stored at -80˚C until further analysis. Lysozyme activity was analysed according to [37] and expressed as U/mol. Lysozyme activity was measured on a Varioskan Flash plate reader (ThermoFisher Scientific, MA, USA), and 250 μL of suspension of Micrococcus lysodeikticus in 0.4 M sodium phosphate buffer at pH 5.8 was added to 5 μL of blood sample, and the absorbance was followed for 30 min. C-reactive protein (CRP) analysis was carried out using a Konelab 30i (ThermoFisher Scientific, MA, USA) and a CRP Plus kit (ThermoFisher Scientific, MA, USA) for the

determination of CRP levels in plasma samples, following an immunoturbidimetric method, and in accordance to the manufacture instructions. Specific antiserum (CRP Plus Antiserum) was added in excess to buffered samples. The absorbance was measured at 340 nm when the reaction has reached the endpoint. The change in absorbance is proportional to the amount of antigen (CRP) in solution, expressed as mg/L.

Fish were analysed per diet group for each sampling point (T_0, n = 20; T_3, n = 30).

Statistical analysis

GraphPad prism 9 (GraphPad software LLC, San Diego, USA) was used for the statistical analysis. Normality distribution of the data was assessed with Shapiro-Wilks test. Normally distributed data were analysed using one-way ANOVA. When the data were not normally distributed, the non-parametric Kruskal-Wallis test was used. A post-hoc (Tukey's multiple comparisons test) was performed to identify which groups differ from each other. Asterisks denote the level of a statistical significance (* = $p < 0.05$). Raw data are provided in supplementary materials (Appendix A).

Results

In vitro study

Cell viability assay. A significant decrease in cell viability was observed only at high tested concentrations (Fig 1) at: 2000 μg/mL for arginine; at 500 and 1000 μg/mL for β-glucan; at 1000 and 2000 μg/mL for PFA 1, and at 100 and 1000 μg/mL for PFA 2. Only vitamin C had values similar to the control at all tested concentrations.

Reactive oxygen species production assay. A significant decrease in ROS production was recorded in cells exposed to vitamin C at all tested concentrations (from 0.1 to 5000 μg/mL), to PFA1 at the lowest concentrations (0.0001 and 0.001 μg/mL) and PFA2 in a range of concentrations from 0.01 to 100 μg/mL (Fig 2). A significant increase in ROS production was observed after exposure to arginine (1 and 100 μg/mL), PFA1 at the highest concentrations (1000 and 2000 μg/mL), and β-glucan at all tested concentrations (from 0.01 to 1000 μg/mL).

Amoebae survival. A significant decrease in the survival of *P. perurans* was observed after exposure to arginine (10000 μg/mL and 1000 μg/mL), PFA1 (10000 μg/mL, 1000 μg/mL) and PFA2 (10000 μg/mL,1000 μg/mL and 100 μg/mL) (Fig 3).

Field trial

Fish growth and survival. There were no statistical differences in the feeding rate and the calculated SGR between groups during the study. The mean SGR percentage was higher in the Protec Gill group (0.73%) compared to the Express group (0,61%), although without significant differences.

Mortality was recorded for a broad period of time (from 3 weeks before the trial (T_{-3}) until 3 weeks after the trial (T_6)) and the calculated average mortality ranged from 0.1% to 0.36% during the whole period (S3 Fig). In terms of growth and mortality rate, the response of the fish to the diets throughout the study was similar, without statistical significancy between diet groups.

Macroscopic gill score. No statistical differences between diet groups were found either in AGD-score or PGD-score after three weeks of administration (Fig 4). However, a progression for both scores was observed at T_3 in both groups. For AGD-score, Protec Gill group showed scores from 0 to 2 at T_0, and from 0 to 3 at T_3 (Fig 4A). Whereas for PGD score,

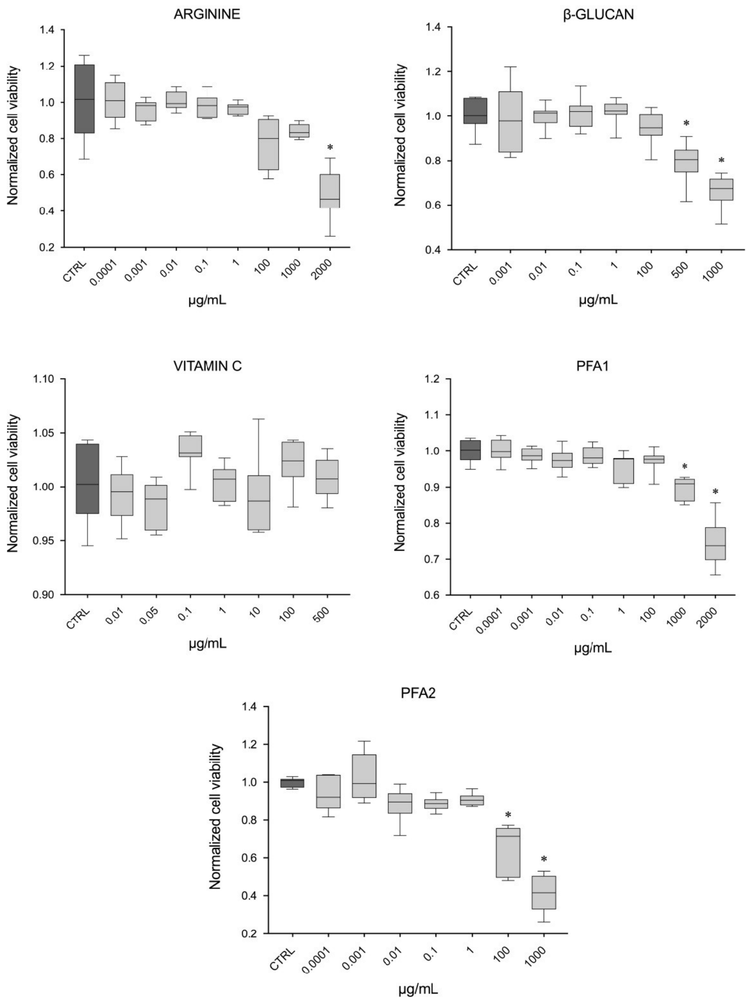

Fig 1. Cell viability. *In vitro* cell viability assay results reported per ingredient: arginine, β-glucan, vitamin C, phytogenic feed additive 1 (PFA1), phytogenic feed additive 2 (PFA2). Values are expressed as µg/mL and reported as median ± standard deviation, normalized to the control (CTRL); (n = 7), * = $p < 0.05$ (compared to the control).

https://doi.org/10.1371/journal.pone.0304112.g001

Express group showed all the five scores at T_3, and Protec Gill group had score from 0 to 4 at T_3 (Fig 4B).

Pathogen detection. A significant decrease in pathogen load (higher Ct value) was recorded for *Ca. B. cysticola* in the Protec Gill group at T_3 compared to the Express group at the same timepoint (Fig 5). No statistical differences were recorded for *P. perurans* and *P. theridion* detection when comparing the two groups. As for SGPV presence, in the Protec Gill group at T_3 there was a lower number of fish in which the virus was detected (3% prevalence, only one fish with high Ct value), compared to the Express group at the same timepoint (13% prevalence). Since SGPV was detected in only one fish in the Protec Gill group, no statistical analysis was carried out.

Gill histology. There was a significant reduction in all the analysed gill lesions (epithelial and mucous hyperplasia, lamellar fusion, necrosis, oedema, and pathogen load) in the Protec Gill group at T_3 compared to the Express group (Fig 6). For epithelial and mucous hyperplasia and lamellar fusion, 50% of the investigated fish population had no lesions (score 0) in the Protec Gill group at T_3 compared to the Express group (Fig 6A and 6B). A

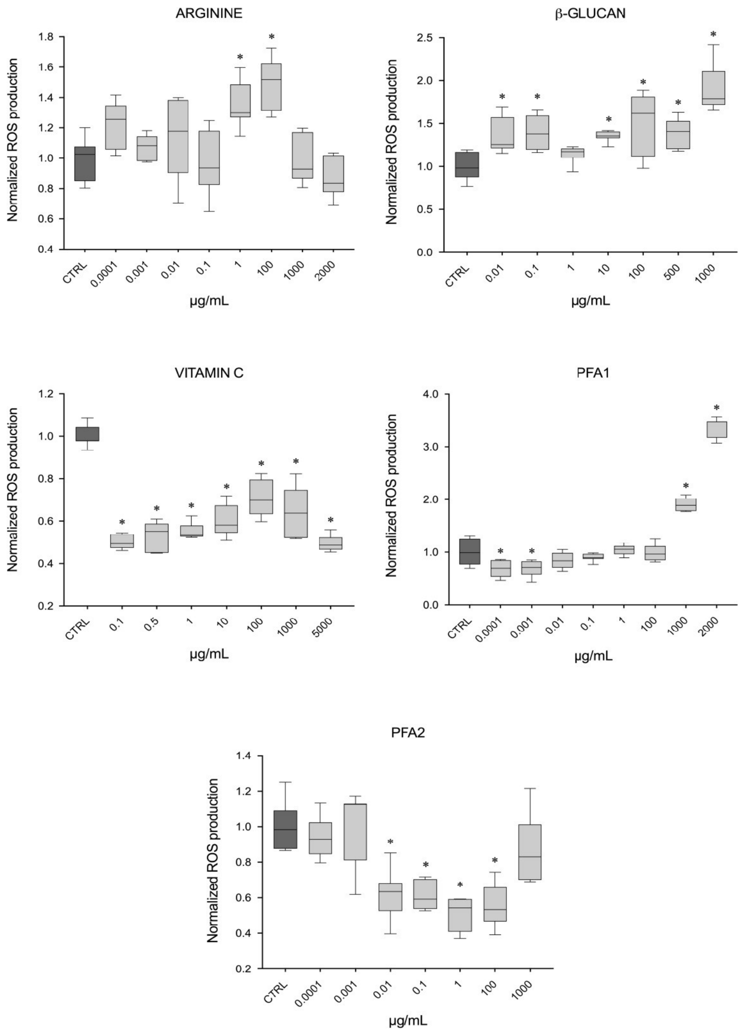

Fig 2. Reactive oxygen species production. ROS production assay, results reported per ingredient: arginine, β-glucan, vitamin C, phytogenic feed additive 1 (PFA1), phytogenic feed additive 2 (PFA2). Values are expressed as μg/mL and reported as median ± standard deviation, normalized to the control (CTRL); (n = 7), * $p < 0.05$ (compared to the control).

https://doi.org/10.1371/journal.pone.0304112.g002

significantly lower number of fish (7%) had mild necrotic changes (score 1) at T_3 in the Protec Gill group compared to the Express group, which had lesions of all three severities (score 1 to 3) (Fig 6C). Interestingly, Protec Gill group showed less necrosis at T_3 compared to T_0, which showed necrosis of all three severities (score 1 to 3). Moreover, at T_3 there was no evidence of oedema or fluid accumulation in the gill tissue of fish in the Protec Gill group compared to the Express group (Fig 6D). The pathogen load, defined as presence of bacteria (epitheliocystis) and parasites (amoebae) in gills, decreased significantly in the Protec Gill group at T_3 compared to the Express group at the same timepoint, and compared to the Protec Gill group at T_0 (Fig 6E).

Plasma parameters. A significant decrease in the lysozyme activity was observed in the Protec Gill group at T_3 compared to the Express group at the same timepoint and compared to both groups at T_0 (Fig 7). No statistical differences were recorded in the CRP values between groups at any timepoint.

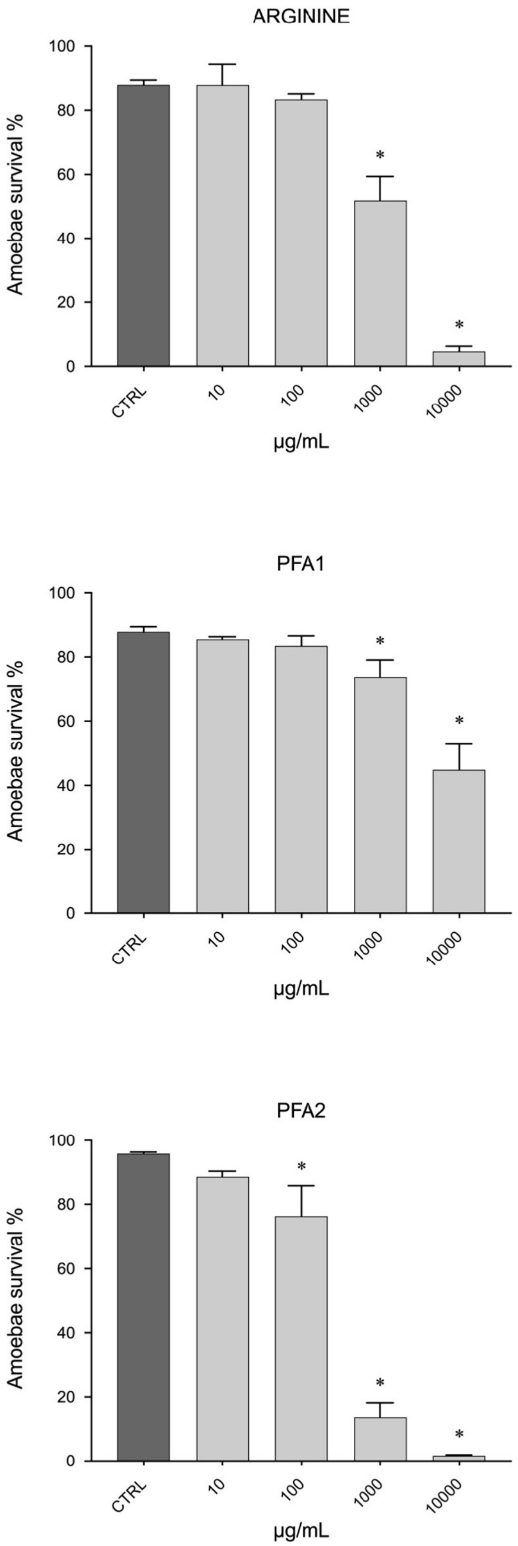

Fig 3. Amoebae survival. Amoebae (*Paramoeba perurans*) survival testing, results reported per ingredient: arginine, phytogenic feed additive 1 (PFA1), phytogenic feed additive 2 (PFA2). Values are expressed as amoebae survival percentage (%) and reported as mean ± standard deviation, compared to the control (CTRL); (n = 3), * $p < 0.05$ (compared to the control).

https://doi.org/10.1371/journal.pone.0304112.g003

A)

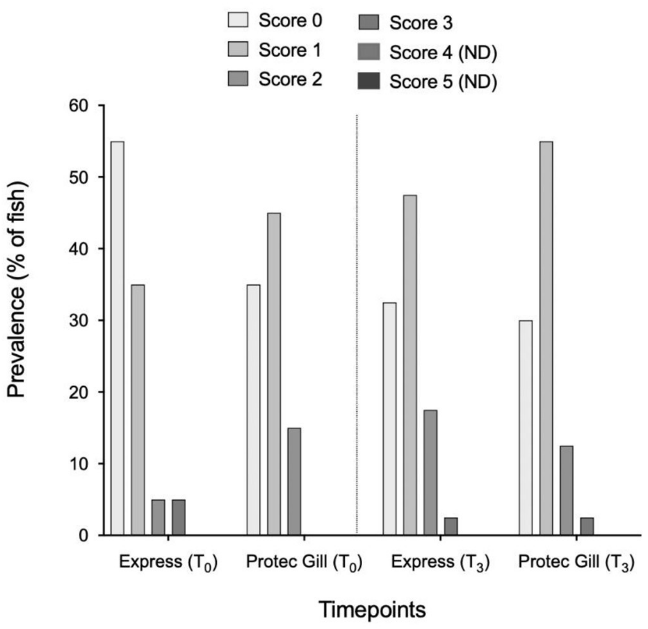

B)

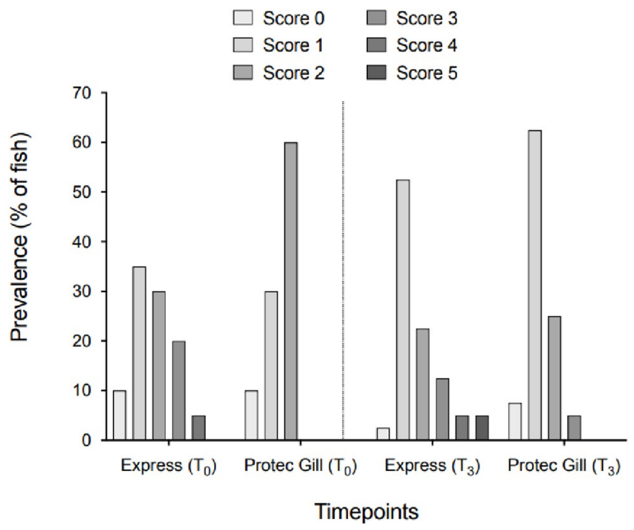

Fig 4. Macroscopic gill score. Amoebic gill disease score (A) and proliferative gill disease score (B) at the beginning of the study (T_0) and at the end (T_3) in Atlantic salmon (*Salmo salar*) gill tissue. Results are reported as prevalence percentage of fish with a specific score; n = 20 (T_0), n = 40 (T_3); ND = not detected.

https://doi.org/10.1371/journal.pone.0304112.g004

Discussion

In vitro cell cultures are an important part of modern research, as they represent a good alternative to *in vivo* testing [38]. Cell lines derived from fish is a promising tool for studying many of the aquaculture challenges such as fish growth, disease assessment and reproduction [39–41]. They also can be effectively used as model systems to investigate nutrient assimilation and

A)

Paramoeba perurans		
**Groups**	**n° of fish**	**Prevalence**
Express T_0	20	100%
Protec Gill T_0	20	100%
Express T_3	30	100%
Protec Gill T_3	30	100%

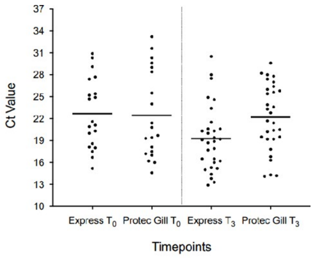

B)

Paranucleospora theridion		
**Groups**	**n° of fish**	**Prevalence**
Express T_0	20	100%
Protec Gill T_0	20	100%
Express T_3	30	100%
Protec Gill T_3	30	100%

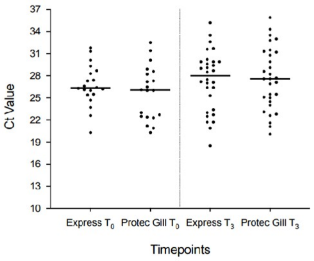

C)

Ca. Branchiomonas cysticola		
**Groups**	**n° of fish**	**Prevalence**
Express T_0	20	100%
Protec Gill T_0	20	100%
Express T_3	30	100%
Protec Gill T_3	30	100%

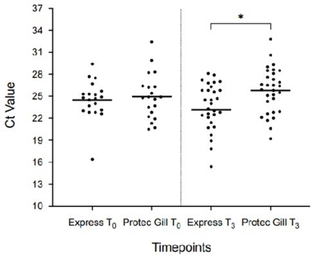

D)

Salmon gill poxvirus		
**Groups**	**n° of fish**	**Prevalence**
Express T_0	20	30%
Protec Gill T_0	20	30%
Express T_3	30	13%
Protec Gill T_3	30	3%

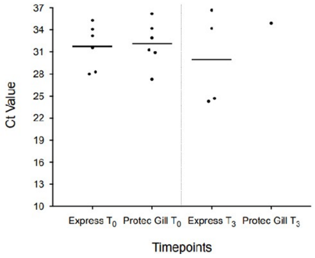

Fig 5. Pathogen detection. Detection of *Paramoeba perurans* (A), *Paranucleospora theridion* (B), *Ca. Branchiomonas cysticola* (C), and salmon gill poxvirus (D), by qPCR, at the beginning of the study (T_0) and at the end (T_3) in Atlantic salmon (*Salmo salar*) gill tissue. Results are reported as mean and individual values; n = 20 (T_0), n = 30 (T_3). Ct value = cycles to threshold value, * $p < 0.05$.

https://doi.org/10.1371/journal.pone.0304112.g005

metabolism, but rarely have been used to study aspects of fish nutrition [39]. Most of the *in vitro* studies on fish nutrition are using the intestinal rainbow trout epithelial cell line (RTgutGC) [42–44], for understanding the functional immunity system of the fish gut as well as the effects of functional feed ingredients in the gut cells [42]. However, in the case of CGD, the use of gill cells seems to be a more appropriate tool to assess the beneficial effects of functional ingredients. Since both gut and gill fish cell lines from Atlantic salmon are not well established as *in vitro* system and are not commercially available, in the current study the suitability of RTgill-W1 cell line was investigated.

Based on the cell viability assay, thresholds of the ingredients' concentration were identified. When applied at high concentrations, all tested functional ingredients, except vitamin C,

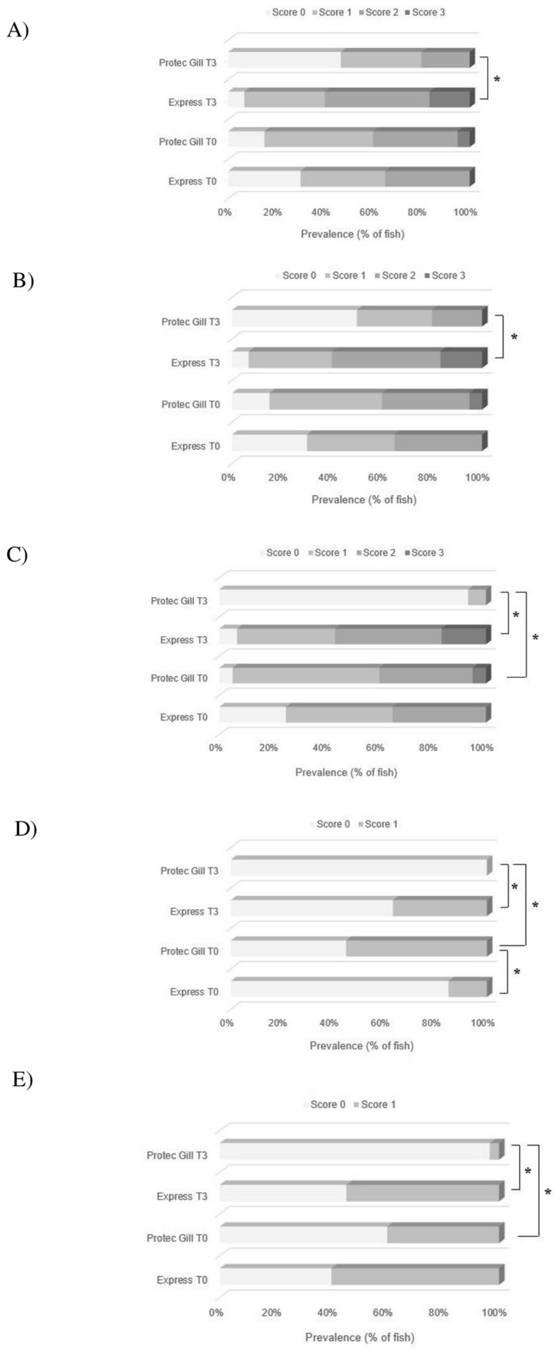

Fig 6. Gill histology. Histopathological analysis of gill lesions analysed in Atlantic salmon gill tissue at the beginning of the study (T_0) and at the end (T_3): (A) epithelial and mucous hyperplasia, (B) lamellar fusion, (C) tissue degeneration/necrosis, (D) oedema, (E) pathogen load. Results are reported as prevalence percentage of fish with a specific score (from 0 to 3 for epithelial and mucous hyperplasia, lamellar fusion, and tissue degeneration/necrosis, and as absence (score 0) and presence (score 1) for oedema and pathogen load); n = 20 (T_0), n = 30 (T_3), * $p < 0.05$.

https://doi.org/10.1371/journal.pone.0304112.g006

significantly reduced cell viability. A clear dose-response curve was observed, where increasing the concentration decreases the viability, probably associated with a decrease in metabolic activity. Furthermore, these results contributed to the identification of the ideal ingredient

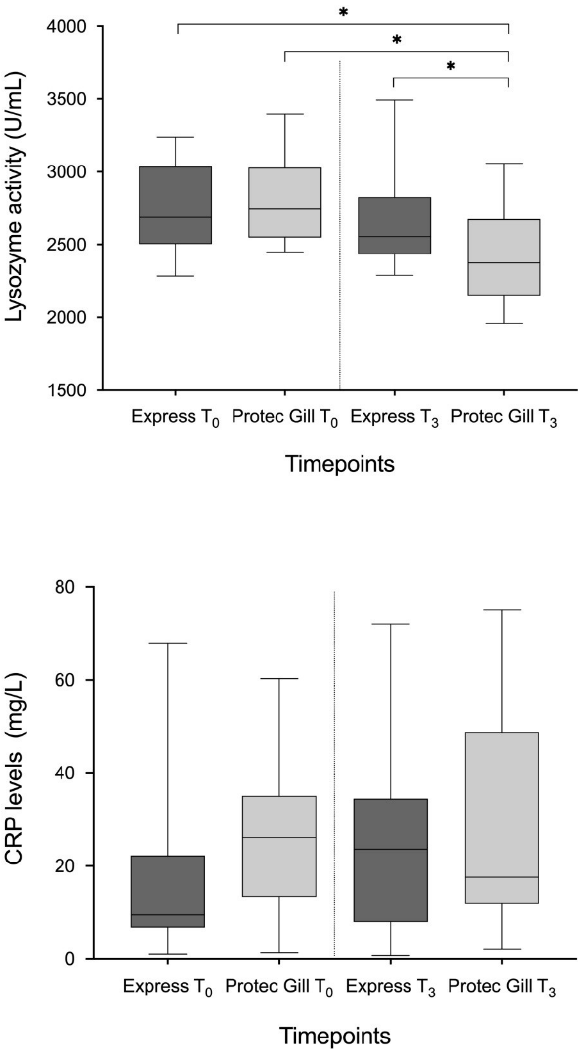

Fig 7. Plasma parameters. Lysozyme expressed as U/mL, and C-reactive protein (CRP) expressed as mg/L, activities results in Atlantic salmon (*Salmo salar*) plasma at the beginning of the study (T_0) and at the end (T_3). Results are reported as median ± standard deviation; n = 20 (T_0), n = 30 (T_3), * $p < 0.05$.

https://doi.org/10.1371/journal.pone.0304112.g007

concentration ranges, which might be useful in establishing the concentration to be included in future diets.

Concerning the production of ROS, the effects differed between ingredients. Arginine and β-glucan showed an increase in ROS production, which was significant at 1–100 μg/mL for arginine and between 0.01 and 1000 μg/mL for β-glucan. According to previous study, β-glucan enhance non-specific host defense mechanisms, stimulating leukocytes to trigger their phagocytic reactions, through the production of ROS [10], which can explain the present study findings. Regardless the role of this ingredient, the highest tested concentrations resulted in a very high ROS level, which are thought to be cytotoxic. This is supported by the cell viability results, which showed a significant reduction in cell survival. Instead, no improvements were reported at cellular level when RTgill-W1 cells were exposed to arginine, used as single ingredient. In fish, antioxidant effects of arginine have been previously evaluated [45–48], but no significant effects were found, in accordance with the current study's findings. However, arginine beneficial contribution might be higher when included in diet formulation, due to

positive interaction with other ingredients. Interestingly, in our study, when amoebae were exposed to arginine, a clear amoebicidal activity at the highest concentrations (10000 and 1000 μg/mL) was observed, highlighting a potential role of arginine to prevent microbial infections, but not at cell viability conditions, where the effects of arginine appears to be less effective on the amoebae survival. This feature has not yet been thoroughly explored and further research is needed to understand the mechanisms behind this aspect.

Vitamin C, as widely reported [49–51], mainly exerted antioxidant properties, showing a significant decrease in ROS production at all tested concentrations (0.1 to 5000 μg/mL). According to a previous study, this beneficial effect is exerted at low concentration, like 0.1 μg/mL, which could be safely used in future fish feed formulations [49].

Two PFAs were tested for assessing their potential as antioxidant and as amoebicidal agents to prevent microbial infections. The PFA1 showed a significant increase in ROS production at concentrations higher than 1000 μg/mL, highlighting a potential cytotoxic effect if provided at high dosage. At low concentrations (0.0001 and 0.001 μg/mL) showed instead a significant decrease in ROS production and might be therefore used for its antioxidant properties. In a concentration range of 0.01 to 100 μg/mL, the second phytogenic, PFA2, significantly reduced the production of ROS, recommending its usage in this range for exerting antioxidant properties. According to a previous study, increasing the level of PFAs is not associated with improvements of growth performance and feed utilization efficiency [52], which can explain the present study findings, where both PFAs exerted antioxidant properties at low dosages. Regarding their potential amoebicidal activity, a significant decrease in the survival of amoebae was observed for both tested PFAs, especially for PFA2 which also showed a significant decrease at the lowest tested concentration (100 μg/mL). According to previous studies [3, 13, 15], PFAs are recognized to have antibacterial, antiviral, antifungal properties and, sometimes, immune stimulatory and antimicrobial effects in fish. Based on the present study, PFA1 and PFA2 showed a clear amoebicidal activity at the highest tested concentrations. However, those ingredients did not display any significant amoebicidal effect within the optimal concentration range for the cell viability. Nevertheless, when included in a diet, they could still contribute positively to reduce the pathogen load, as suggested by the *in vivo* trial results. PFAs have been recently introduced in aquaculture, therefore their potential is not deeply explored yet, and additional research is needed to assess their ability as single ingredients and in a mixture in both *in vitro* and *in vivo* studies. Implementing an *in vitro* cell-based approach, using multiple assays aimed at measuring different endpoints, various cell line types, and innovative strategies, such as co-culturing of different cell lines, might be helpful to understand the limits related to standard culture conditions. This also represents a promising alternative to reduce the current dependence from fish trials for screening functional ingredients and diets.

Currently available methods for preventing and treating fish diseases are expensive and shown limited efficacy [53–54]. Moreover, as no vaccines nor specific treatments are licensed to treat CGD, the optimization of diet formulation might be a successful strategy to improve fish health and decrease disease susceptibility [17]. Herein, the beneficial effects of a commercially available functional feed, Protec Gill, were investigated in fish affected by CGD.

The most effective, non-invasive, and commonly used means for the assessment of gill diseases on a commercial scale is carried out through the gross pathological evaluation of gill arches to identify focal/multifocal lesions [55–56], and through the evaluation of growth and mortality rates. Although, these methods might not be sensitive enough to show fish health improvements during feeding experiments, as in the current study. Herein, fish affected by CGD fed with two different diets had similar AGD and PGD scores after three weeks, and neither mortality nor growth showed significant differences between diet groups. However, a clear improvement in gill tissue was observed through the histopathological assessment. The

selected histological parameters, epithelial and mucous hyperplasia, lamellar fusion, tissue degeneration/necrosis, oedema, and pathogen load, are known to be the most representative lesions during gill disease outbreaks [56]. A significant improvement in gill tissue after three weeks of Protec Gill feeding was demonstrated, with a significant reduction of all the analysed gill lesions. Interestingly, in the Protec Gill group after three weeks there was a significant reduction of epithelial and mucous hyperplasia (50% of fish had score 0), which is reported to be one of the predominant characteristics in fish affected by CGD [25], and a significant reduction of pathogen load, in particular the presence of epitheliocystis, which is associate with the presence of *Ca. B. cysticola* [57], one of the major contributors of CGD [23]. The analysis of plasma parameters, especially the lysozyme activity, also confirmed a reduction in the infection of fish after three weeks of Protec Gill feeding. CRP levels were not able to distinguish group conditions after three weeks, probably due to the fact that is more responsive during acute inflammatory episodes [58].

In literature, most of the studies are focused on describing the mechanisms behind the development of CGD, the role of each pathogen involved, and the use of histopathological gill assessment as a more in-depth investigation [23–26, 59–60]. However, one aspect that has not been yet deeply explored is the investigation of alternative strategies to improve gill tissue conditions and fish health during CGD outbreaks, especially in absence of vaccines or specific treatments. Our study findings suggests that the use of functional diets is a feasible strategy for preventing or mitigating the increasingly common gill diseases, particularly in cases of CGD, and further research could focus on evaluating the use of such functional diets for preventing disease outbreaks.

Conclusion

In fish, knowledge about basic mode of action of functional ingredients and their interactions when included in a diet is weak and fragmentary. The *in vitro* model, which may be implemented with additional assays and cell line types, appears to be a suitable alternative for screening ingredients, reducing the number of *in vivo* trials for a more sustainable aquaculture practice.

The use of a diet containing selected functional feed ingredients in a field trial was a successful strategy for mitigating the CGD, by slowing disease progression, reducing pathogen load, and significantly improving gill tissue condition.

Supporting information

S1 Table. Functional feed ingredients. Functional feed ingredients used for the *in vitro* assays. (A) RTgill-W1 assays; (B) Amoebae survival testing.
(DOCX)

S2 Table. Field trial. Estimated average fish weight (Kg) and number of fish in each cage at the beginning of the study (T_0).
(DOCX)

S3 Table. Diet formulations. Formulation and proximate composition of the feed used in the field trial.
(DOCX)

S4 Table. Delousing events. Overview of delousing events of the different cages before and under trial period. Period numbers indicate weeks before and during the trial.
(DOCX)

S5 Table. Histopathological gill score. Scoring system of the histopathological gill lesions analysed, adapted from [36].
(DOCX)

S1 Fig. Images scoring system. Representative images of the semiquantitative scoring system described in S5 Table, represents gill histopathology as low (A, C, E and G) and high (B, D, F and H) power view. Score 0: none to focal lesions such as clubbing (arrow) and fusion. Score 1: (C) mild multifocal areas of lesions (arrows) comprise of epithelial hyperplasia, fusion, and inflammation (D) (star). Score 2: (E) moderate multifocal patches of lesions (arrow) and (F) vesicle formation. Score 3: (G) severe widespread increase in lamellar hyperplasia/fusion and (H) inflammation (arrow). Reprinted from Dr. Naveed M. Yousaf under a CC BY license, with permission from PLOS ONE, original copyright 2024.
(DOCX)

S2 Fig. Images gill lesions. Histological sections of gills from Atlantic salmon (Salmo salar) stained with haematoxylin and eosin. Representative image showing: (A) amoeba (arrow) surrounding the secondary lamella; (B) multiple micro-cysts (epitheliocystis, arrow) in the epithelial cells as regular round to oval could be seen in the secondary lamella; (C) epithelial hyperplasia and fusion of secondary lamella with inflammation (star) were noted; (D) epithelial and mucous hyperplasia with fusion could be seen; (E) focal areas of necrosis with vacuolization (star). Reprinted from Dr. Naveed M. Yousaf under a CC BY license, with permission from PLOS ONE, original copyright 2024.
(DOCX)

S3 Fig. Mortality. Weekly mortality relative to the fish count in each group. Timepoints are reported in weeks starting from three weeks prior to the trial (T_{-3}) until three weeks post-trial (T_6).
(DOCX)

Acknowledgments

We gratefully acknowledge the farming company, Bremnes Seashore AS, for the development of the field trial, Dr. Astrid Holzer from the Institute of Parasitology, Biology Centre of Czech Academy of Science (Czech Republic) for the work with *Paramoeba perurans*, and the engineers at the University of Stavanger for technical support.

Author Contributions

Conceptualization: Matteo Vitale, Eirik Hoel, Muhammad Naveed Yousaf, Martha Amalie Kambestad, Julia Mullins, Leidy Lagos, Kjetil Berge, Charles McGurk, Daniela Maria Pampanin.

Data curation: Matteo Vitale, Eirik Hoel, Muhammad Naveed Yousaf, Martha Amalie Kambestad, Julia Mullins, Leidy Lagos, Kjetil Berge, Daniela Maria Pampanin.

Formal analysis: Matteo Vitale, Muhammad Naveed Yousaf.

Funding acquisition: Matteo Vitale, Leidy Lagos, Charles McGurk, Daniela Maria Pampanin.

Supervision: Leidy Lagos, Daniela Maria Pampanin.

Writing – original draft: Matteo Vitale.

Writing – review & editing: Eirik Hoel, Muhammad Naveed Yousaf, Julia Mullins, Kjetil Berge, Charles McGurk, Daniela Maria Pampanin.

References

1. Bostock J, McAndrew B, Richards R, Jauncey K, Telfer T, Lorenzen K, et al. Aquaculture: global status and trends. Philosophical transaction of the royal society B: Biological Sciences. 2010; 365(1554), 2897–2912. https://doi.org/10.1098/rstb.2010.0170 PMID: 20713392
2. Olmos Soto J, Paniagua-Michel JDJ, Lopez L, Ochoa L. Functional feeds in aquaculture. Springer handbook of marine biotechnology. 2015; 1303–1319
3. Dawood MA, Koshio S, Esteban MA. Beneficial roles of feed additives as immunostimulants in aquaculture: a review. Reviews in Aquaculture. 2018; 10(4), 950–974. https://doi.org/10.1111/raq.12209
4. Kiron V. Fish immune system and its nutritional modulation for preventive health care. Animal Feed Science and Technology. 2012; 173(1–2), 111–133. https://doi.org/10.1016/j.anifeedsci.2011.12.015
5. Chen G, Liu Y, Jiang J, Jiang W, Kuang S, Tang L, et al. Effect of dietary arginine on the immune response and gene expression in head kidney and spleen following infection of Jian carp with *Aeromonas hydrophila*. Fish & Shellfish Immunology. 2015; 44(1), 195–202. https://doi.org/10.1016/j.fsi.2015.02.027
6. Lin H, Tan X, Zhou C, Niu J, Xia D, Huang Z, et al. Effect of dietary arginine levels on the growth performance, feed utilization, non-specific immune response, and disease resistance of juvenile golden pompano *Trachinotus ovatus*. Aquaculture. 2015; 437, 382–389. https://doi.org/10.1016/j.aquaculture.2014.12.025
7. Hamre K, Sissener NH, Lock EJ, Olsvik PA, Espe M, Torstensen BE, et al. Antioxidant nutrition in Atlantic salmon (*Salmo salar*) parr and post-smolt, fed diets with high inclusion of plant ingredients and graded levels of micronutrients and selected amino acids. PeerJ. 2016; 4, e2688. https://doi.org/10.7717/peerj.2688 PMID: 27843721
8. Tewary A, Patra BC. Use of vitamin C as an immunostimulant. Effect on growth, nutritional quality, and immune response of *Labeo rohita* (Ham.) Fish physiology and biochemistry. 2008; 34(3), 251–259. https://doi.org/10.1007/s10695-007-9184-z PMID: 18665463
9. Misra CK, Das BK, Mukherjee SC, Pattnaik P. Effect of long term administration of dietary β-glucan on immunity, growth, and survival of *Labeo rohita* fingerlings. Aquaculture. 2006; 255(1–4), 82–94. https://doi.org/10.1016/j.aquaculture.2005.12.009
10. Hadiuzzaman M, Moniruzzaman M, Shahjahan M, Bai SC, Min T, Hossain Z. Beta-glucan: mode of action and its uses in fish immunomodulation. Frontiers in Marine Science. 2022; 1179. https://doi.org/10.3389/fmars.2022.905986
11. Kamilya D, Maiti TK, Joardar SN, Mal BC. Adjuvant effect of mushroom glucan and bovine lactoferrin upon *Aeromonas hydrophila* vaccination in catla, *Catla catla* (Hamilton). Journal of Fish Diseases. 2006; 29(6), 331–337. https://doi.org/10.1111/j.1365-2761.2006.00722.x PMID: 16768713
12. Borges A, Abreu AC, Dias C, Saavedra MJ, Borges F, Simões M. New perspective on the use of phytochemicals as an emergent strategy to control bacterial infections including biofilms. Molecules. 2016; 21(7), 877. https://doi.org/10.3390/molecules21070877
13. Bulfon C, Volpatti D, Galeotti M. Current research on the use of plant-derived products in farmed fish. Aquaculture research. 2015; 46(3), 513–551. https://doi.org/10.1111/are.12238
14. Stratev D, Zhelyazkov G, Noundou XS, Krause RW. Beneficial effects of medicinal plants in fish diseases. Aquaculture International. 2018; 26, 289–308. https://doi.org/10.1007/s10499-017-0219-x
15. Yang C, Chowdhury MK, Hou Y, Gong J. Phytogenic compounds as alternatives to in-feed antibiotics: potential and challenges in application. Pathogens. 2015; 4(1), 137–156. https://doi.org/10.3390/pathogens4010137 PMID: 25806623
16. Martinez-Rubio L, Morais S, Evensen Ø, Wadsworth S, Ruohonen K, Vecino JL, et al. Functional feeds reduce hearth inflammation and pathology in Atlantic salmon (*Salmo salar*) following experimental challenge with Atlantic salmon reovirus (ASRV). PloS one. 2012; 7(11), e40266. https://doi.org/10.1371/journal.pone.0040266
17. Talbot A, McCormack M, Dwivedi A, O'Connor I, Valdenegro V, MacCharthy E. Investigation into the potential use of dietary supplementation to reduce the impact of Amoebic Gill Disease. Aquaculture. 2022; 552, 737983. https://doi.org/10.1016/j.aquaculture.2022.737983
18. Rozas-Serri M. Gill diseases in marine salmon aquaculture with an emphasis on amoebic gill disease. CABI Reviews. 2019; 1–15. https://doi.org/10.1079/PAVSNNR201914032
19. Gunnarsson GS, Blindheim S, Karlsbakk E, Plarre H, Imsland AK, Handeland S, et al. *Desmozoon lepeophtherii* (microsporidian) infections and pancreases disease (PD) outbreaks in farmed Atlantic

salmon (*Salmo salar* L.). Aquaculture. 2017; 468, 141–148. https://doi.org/10.1016/j.aquaculture.2016.09.035

20. Matthews CGG, Richards RH, Shinn AP, Cox DI. Gill pathology in Scottish farmed Atlantic salmon, *Salmo salar* L., associated with the microsporidian *Desmozoon lepeophtherii*. Freeman et Sommerville. Journal of fish diseases. 2013; 36(10), 861–869. https://doi.org/10.1111/jfd.12084

21. Noguera P, Olsen AB, Hoare J, Lie KI, Marcos-Lopez M, Poppe TT, et al. Complex gill disorder (CGD): A histopathology workshop report. Bulletin of the European Association of Fish Pathologists. 2019; 39 (4).

22. Gjessing MC, Thoen E, Tengs T, Skotheim SA, Dale OB. Salmon gill poxvirus, a recently characterized infectious agent of multifactorial gill disease in freshwater and seawater reared Atlantic salmon. Journal of fish diseases. 2017; 40(10), 1253–1265. https://doi.org/10.1111/jfd.12608 PMID: 28105681

23. Gjessing MC, Splisberg B, Steinum TM, Amundsen M, Austbø L, Hansen H, et al. Multi-agent is situ hybridization confirms *Ca. Branchiomonas cysticola* as a major contributor in complex gill disease in Atlantic salmon. Fish & Shellfish Immunology Reports 2. 2021; 100026. https://doi.org/10.1016/j.fsirep.2021.100026

24. Boerlage AS, Ashby A, Herrero A, Reeves A, Gunn GJ, Rodger HD. Epidemiology of marine gill diseases in Atlantic salmon (*Salmo salar*) aquaculture: a review. Reviews in aquaculture. 2020; 12(4), 2140–2159. https://doi.org/10.1111/raq.12426

25. Gjessing MC, Steinum T, Olsen AB, Lie KI, Tavornpanich S, Colquhoun DJ, et al. Histopathological investigation of complex gill disease in sea farmed Atlantic salmon. PloS One. 2019; 14(10), e0222926. https://doi.org/10.1371/journal.pone.0222926 PMID: 31581255

26. Herrero A, Thompson KD, Ashby A, Rodger HD, Dagleish MP. Complex gill disease: an emerging syndrome in farmed Atlantic salmon (*Salmo salar*). Journal of Comparative Pathology. 2018; 163, 23–28. https://doi.org/10.1016/j.jcpa.2018.07.004

27. Kvellestad A, Falk K, Nygaard SM, Flesjå K, Holm JA. Atlantic salmon paramyxovirus (ASPV) infection contributes to proliferative gill inflammation (PGI) in seawater-reared *Salmo salar*. Diseases of aquatic organisms. 2005; 67(1–2), 47–54. https://doi.org/10.3354/dao067047 PMID: 16385807

28. Nylund A, Watanabe K, Nylund S, Karlsen M, Saether PA, Arnesen CE, et al. Morphogenesis of salmonid gill poxvirus associated with proliferative gill disease in farmed Atlantic salmon (*Salmo salar*) in Norway. Archives of virology. 2008; 153, 1299–1309. https://doi.org/10.1007/s00705-008-0117-7 PMID: 18521535

29. Lee LE, Dayeh VR, Schirmer K, Bols NC. Applications and potential uses of fish gill cell lines: examples with RTgill-W1. In vitro cellular & developmental biology-animal. 2009; 45, 127–134. https://doi.org/10.1007/s11626-008-9173-2 PMID: 19184248

30. Kamiloglu S, Sari G, Ozdal T, Capanoglu E. Guidelines for cell viability assays. Food frontiers. 2020; 1 (3), 332–349. https://doi.org/10.1002/fft2.44

31. LeBel CP, Ischiropoulos H, Bondy SC. Evaluation of the probe 2', 7'-dichlorofluorescin as an indicator of reactive oxygen species formation and oxidative stress. Chemical research in toxicology. 1992; 5(2), 227–231. https://doi.org/10.1021/tx00026a012 PMID: 1322737

32. MacPhail DP, Koppenstein R, Maciver SK, Paley R, Longshaw M, Henriquez FL. Vibrio species are predominantly intracellular within cultures of *Neoparamoeba perurans*, causative agent of Amoebic Gill Disease (AGD). Aquaculture. 2021; 532, 736083. https://doi.org/10.1016/j.aquaculture.2020.736083

33. Taylor RS, Muller WJ, Cook MT, Kube PD, Elliot NG. Gill observations in Atlantic salmon (*Salmo salar* L.) during repeated amoebic gill disease (AGD) field exposure and survival challenge. Aquaculture. 2009; 290(1–2), 1–8. https://doi.org/10.1016/j.aquaculture.2009.01.030

34. Løvoll M, Austbø L, Jørgensen JB, Rimstad E, Frost P. Transcription of reference genes used for quantitative RT-PCR in Atlantic salmon is affected by viral infection. Veterinary research. 2011; 42, 1–5. https://doi.org/10.1186/1297-9716-42-8

35. Haugarvoll E, Bjerkås I, Nowak B, Hordvik I, Koppang EO. Identification and characterization of a novel intraepithelial lymphoid tissue in the gills of Atlantic salmon. Journal of Anatomy. 2008; 213(2), 202–209. https://doi.org/10.1111/j.1469-7580.2008.00943.x PMID: 19172734

36. Herrero A, Rodger H, Hayward AD, Cousens C, Bron JE, Dagleish MP, et al. Prospective longitudinal study of putative agents involved in complex gill disorder in Atlantic salmon (*Salmo salar*). Pathogens. 2022; 11(8), 878. https://doi.org/10.3390/pathogens11080878 PMID: 36014998

37. Mullins J, Nowak B, Leef M, Røn Ø, Eriksen TB, McGurk C. Functional diets improve survival and physiological response of Atlantic salmon (*Salmo salar*) to amoebic gill disease. Journal of the World Aquaculture Society. 2020; 51(3), 634–648. https://doi.org/10.1111/jwas.12692

38. Thangaraj RS, Narendrakumar L, Prasannan Geetha P, Shanmuganathan AR, Dharmaratnam A, Nithianantham SR. Comprehensive update on inventory of finfish cell lines developed during the last

decade (2010–2020). Reviews in Aquaculture. 2021; 13(4), 2248–2288. https://doi.org/10.1111/raq.12566

39. Goswami M, Yashwanth BS, Trudeau V, Lakra WS. Role and relevance of fish cell line in advanced *in vitro* research. Molecular Biology Reports. 2022; 1–19. https://doi.org/10.1007/s11033-021-06997-4

40. Rubio N, Datar I, Stachura D, Kaplan D, Krueger K. Cell-based fish: a novel approach to seafood production and an opportunity for cellular agriculture. Frontiers in Sustainable Food Systems. 2019; 3, 43. https://doi.org/10.3389/fsufs.2019.00043

41. Bols NC. Biotechnology and aquaculture: the role of cell cultures. Biotechnology advances. 1991; 9(1), 31–49. https://doi.org/10.1016/0734-9750(91)90403-I PMID: 14543738

42. Wang J, Lei P, Gamil AAA, Lagos L, Yue Y, Schirmer K, et al. Rainbow trout (*Oncorhynchus mykiss*) intestinal epithelial cells as a model for studying gut immune function and effects of functional feed ingredients. Frontiers in immunology. 2019; 10, 152. https://doi.org/10.3389/fimmu.2019.00152 PMID: 30792715

43. Kawano A, Haiduk C, Schirmer K, Hanner R, Lee LEJ, Dixon B, et al. Development of a rainbow trout intestinal epithelial cell line and its response to lipopolysaccharide. Aquaculture Nutrition. 2011; 17(2), e241–e242. https://doi.org/10.1111/j.1365-2095.2010.00757.x

44. Minghetti M, Drieschner C, Bramaz N, Schug H, Schirmer K. A fish intestinal epithelial barrier model established from the rainbow trout (*Oncorhynchus mykiss*) cell line, RTgutGC. Cell biology and toxicology. 2017; 33, 539–555. https://doi.org/10.1007/s10565-017-9385-x PMID: 28251411

45. Coutinho F, Castro C, Rufino-Palomares E, Ordóñez-Grande B, Gallardo MA, Kaushik S, et al. Dietary arginine surplus does not improve intestinal nutrient absorption capacity, amino acid metabolism and oxidative stress status of gilthead sea bream (*Sparus aurata*) juveniles. Aquaculture. 2016; 464, 480–488. https://doi.org/10.1016/j.aquaculture.2016.07.032

46. Ren M, Liao Y, Xie J, Liu B, Zhou Q, Ge X, et al. Dietary arginine requirement of juvenile blunt snout bream, *Megalobrama amblycephala*. Aquaculture. 2013; 414, 229–234. https://doi.org/10.1016/j.aquaculture.2013.08.021

47. Zhou H, Chen N, Qiu X, Zhao M, Jin L. Arginine requirement and effect of arginine intake on immunity in largemouth bass, *Micropterus salmoides*. Aquaculture Nutrition. 2012a; 18, 107–116. https://doi.org/10.1111/j.1365-2095.2011.00886.x

48. Hoseini SM, Ahmad Khan M, Yousefi M, Costas B. Roles of arginine in fish nutrition and health: insights for future researches. Reviews in Aquaculture. 2020; 12(4), 2091–2108. https://doi.org/10.1111/raq.12424

49. Leal E, Zarza C, Tafalla C. Effect of vitamin C on innate immune responses of rainbow trout (*Oncorhynchus mykiss*) leukocytes. Fish & Shellfish Immunology. 2017. 67, 179–188. https://doi.org/10.1016/j.fsi.2017.06.021 PMID: 28602736

50. Hamre K, Sissener NH, Lock EJ, Olsvik PA, Espe M, Torstensen BE, et al. Antioxidant nutrition in Atlantic salmon (*Salmo salar*) parr and post-smolt, fed diets with high inclusion of plant ingredients and graded levels of micronutrients and selected amino acids. PeerJ. 2016; 4, e2688. https://doi.org/10.7717/peerj.2688 PMID: 27843721

51. Tewary A, Patra BC. Use of vitamin C as an immunostimulant. Effect on growth, nutritional quality, and immune response of *Labeo rohita* (Ham.). Fish physiology and biochemistry. 2008; 34(3), 251–259. https://10.1007/s10695-007-9184-z

52. Abo-State HA, El-Monairy MM, Hammouda YA, Elgendy MY. Effect of phytogenic feed additive on the growth performance and susceptibility of *Oreochromis niloticus* to *Aeromonas hydrophila*. Journal of fisheries and aquatic science. 2017; 12, 141–148. https://doi.org/10.3923/jfas.2017.141.148

53. Marcos-Lopez M, Rodger HD. Amoebic gill disease and host response in Atlantic salmon (*Salmo salar* L.): A review. Parasite immunology. 2020; 42(8), e 12766. https://doi.org/10.1111/pim.12766 PMID: 32564378

54. Oldham T, Rodger H, Nowak BF. Incidence and distribution of amoebic gill disease (AGD)–An epidemiological review. Aquaculture. 2016; 457, 35–42. https://doi.org/10.1016/j.aquaculture.2016.02.013

55. Adams MB, Ellard K, Nowak BF. Gross pathology and its relationship with histopathology of amoebic gill disease (AGD) in farmed Atlantic salmon, *Salmo salar* L. Journal of Fish Diseases. 2004; 27(3), 151–161. https://doi.org/10.1111/j.1365-2761.2004.00526.x PMID: 15009241

56. Clark A, Nowak BF. Field investigations of amoebic gill disease in Atlantic salmon, *Salmo salar* L., in Tasmania. Journal of Fish Diseases. 1999; 22(6), 433–443. https://doi.org/10.1046/j.1365-2761.1999.00175.x

57. Toenshoff ER, Kvellestad A, Mitchell SO, Steinum T, Falk K, Colquhoun DJ, et al. A novel betaproteobacterial agent of gill epitheliocystis in seawater farmed Atlantic salmon (*Salmo salar*). PloS one. 2012; 7(3), e32696. https://doi.org/10.1371/journal.pone.0032696 PMID: 22427865

58. Pepys MB, Booth SE, Tennent GA, Butler PJG, Williams DG. Binding of pentraxins to different nuclear structures: C-reactive protein binds to small nuclear ribonucleoprotein particles, serum amyloid P component binds to chromatin and nucleoli. Clinical & Experimental Immunology. 1994. 97(1), 152–157. https://doi.org/10.1111/j.1365-2249.1994.tb06594.x

59. Østevik L, Stormoen M, Helberg H, Kraugerud M, Manji F, Lie KI, et al. A cohort study of gill infections, gill pathology and gill-related mortality in sea-farmed Atlantic salmon (*Salmo salar* L.): A descriptive analysis. Journal of fish diseases. 2022; 45(9), 1301–1321. https://doi.org/10.1111/jfd.13662

60. Nowak BF, Carson J, Powell MD, Dykovà I. Amoebic gill disease in the marine environment. Bulletin-European Association of Fish Pathologists. 2002; 22(2), 144–147. https://hdl.handle.net/102.100.100/595443
